# Unveiling Immune System Perturbations in Early Development Through Zebrafish Models of NADHX Repair Deficiency

**DOI:** 10.1002/jimd.70149

**Published:** 2026-02-01

**Authors:** Myrto Patraskaki, Najmesadat Seyedkatouli, Lisa Schlicker, Marc O. Warmoes, Maria Lorena Cordero‐Maldonado, Ursula Heins‐Marroquin, Carole L. Linster

**Affiliations:** ^1^ Luxembourg Centre for Systems Biomedicine University of Luxembourg Esch‐sur‐Alzette Luxembourg

**Keywords:** immune system, inborn errors of metabolism, metabolite repair, NAXD, NAXE, zebrafish

## Abstract

The vital cofactors NADH and NADPH are prone to hydration, forming hydroxylated redox‐inactive derivatives (NADHX and NADPHX) in cells. These damaged metabolites are repaired by two highly conserved enzymes, an NAD(P)HX dehydratase (NAXD) and an NAD(P)HX epimerase (NAXE). Mutations in *NAXE* or *NAXD* cause early onset progressive encephalopathy (PEBEL1 or PEBEL2), typically induced by fever or other triggers, and leading to premature death. To advance our comprehension of the disease mechanism and investigate potential therapeutic strategies, we generated zebrafish lines deficient in *naxe* or *naxd* using CRISPR/Cas9 technology. While both models accumulated NADHX, only *naxd* ^*−/−*^ larvae developed a severe phenotype, showing reduced locomotion and early death, which was partially rescued by nicotinic acid supplementation. Both mutant lines displayed signs of dysregulated immune function based on gene expression analyses and increased neutral red staining in the head region, indicating an increased number or activation of microglial cells. Our findings suggest that immune system perturbations play a role in PEBEL disease development, aligning with its inflammatory trigger‐induced nature in humans. The *naxd* ^−/−^ model's responsiveness to nicotinic acid underscores its utility for preclinical drug screening. Overall, these models will be instrumental in furthering our understanding of PEBEL disease mechanisms and enhancing translational research efforts.

## 1 Introduction

Deficiency in the NAD(P)HX repair system, caused by mutations in the *NAXE* or *NAXD* genes, leads to severe neurometabolic disorders known as PEBEL1 (OMIM #617186) and PEBEL2 (OMIM #618321), respectively (Progressive early‐onset Encephalopathy with Brain Edema and/or Leukoencephalopathy) [1, 2]. To date, 45 patients with either *NAXE* or *NAXD* deficiency have been described, of whom more than 70% experienced premature death [3, 4, 5]. The clinical symptoms most often arise in early childhood, primarily including neurological symptoms such as neurodevelopmental regression, seizures, and cognitive impairment, but also skin lesions, hypotonia, and respiratory failure [1, 2, 6, 7, 8, 9, 10, 11, 12, 13, 14, 15, 16, 17, 18, 19, 20, 21, 22]. The disease onset is typically triggered by episodes of immune stress, most commonly benign febrile illness. Immune phenotypes, including recurrent viral infections and elevated inflammatory markers, were also reported for 10 out of 14 surveyed *NAXD* patients [1, 2, 12, 13, 14, 16, 17, 18, 20]. The inflammatory‐triggered disease onset and immune phenotypes suggest a potentially important role for the immune system in the disease pathogenesis.

All patients showed accumulation of NADHX. NADHX and NADPHX are formed by hydration of the nicotinamide ring of the NAD(P)H cofactors and exist in two epimeric forms [23]. S‐ and R‐NAD(P)HX can react further to generate a more stable cyclic end product. None of these damaged derivatives can act as redox cofactors, but they can inhibit NAD(P)‐dependent enzymes [24, 25, 26, 27]. The repair system consists of two highly conserved enzymes, an NAD(P)HX epimerase (NAXE, EC 5.1.99.6) that catalyzes the interconversion of R‐ and S‐NAD(P)HX and an ATP‐dependent S‐NAD(P)HX dehydratase (NAXD, EC 4.2.1.93) that stereospecifically converts S‐NAD(P)HX back to NAD(P)H [23, 28]. Of note, it is the reduced cofactors that are prone to hydration damage, and the repair enzymes act on both NADHX and NADPHX [23]; however, given the even higher instability of NADPHX compared to NADHX, the former is difficult to quantify in biological matrices, and most studies in the field, including the present one, focus on NADHX measurements.

The mechanisms by which NAD(P)H repair deficiency leads to this fatal neurometabolic disorder remain poorly understood and may involve accumulation of damaged NAD(P)HX, deprivation of the physiological cofactors, or a combination thereof. Recent studies reported stabilization and, in some instances, improvement of the clinical outcome in PEBEL patients upon administration of high‐dose vitamin B3 [6, 9, 10, 15, 16], an NAD^+^ precursor, conferring hope that PEBEL disorders are amenable to treatment.

To progress in our understanding of PEBEL disease mechanisms and provide platforms for preclinical therapy development, we aimed here to generate in vivo disease models by knocking out the zebrafish orthologues of the human *NAXD* and *NAXE* genes. Mutant *naxe* and *naxd* larvae showed NADHX accumulation and decreased levels of the healthy NAD cofactor. The *naxd* ^−/−^ larvae spontaneously developed altered locomotor behavior and early lethality, but *naxe* ^−/−^ larvae reached adulthood without gross phenotypes. Since neurological symptoms in PEBEL patients typically emerge following inflammatory triggers and given the reported inflammatory phenotypes in *NAXD* patients, we looked for inflammatory markers in *naxd* ^−/−^ larvae, where neutral red staining and gene expression analyses revealed signs of a pro‐inflammatory state. Despite the absence of an overt phenotype, transcriptomic analyses highlighted the immune response as the most significantly modulated process also in the *naxe* ^*−/−*^ larvae. Importantly, supplementation with nicotinic acid, a B3 vitamer, significantly ameliorated the locomotor defects and increased the survival of *naxd* ^−/−^ larvae. Collectively, the results suggest that NAD(P)HX repair deficiency impairs immune system development and that our zebrafish lines constitute relevant models to further elucidate the molecular basis of *NAXE* and *NAXD* deficiency disorders and to devise therapeutic approaches for PEBEL patients.

## 2 Methods

### 2.1 Animal Husbandry and Handling

Wild‐type (WT) and mutant adult zebrafish lines (all with the AB background) were maintained and fed in the Aquatic Facility of the Luxembourg Centre for Systems Biomedicine according to standard protocols [29]. The original AB stock used for in‐house breeding was a kind gift from the Laboratory of Molecular Biodiscovery at KU Leuven. Zebrafish embryos, collected from natural spawning, from 1 hpf (hours post‐fertilization) to 5 dpf (days post‐fertilization), were maintained at a density of 80–90 embryos in 90 mm Petri dishes in 20–30 mL of 0.3× Danieau's solution (for composition see [30]) in light/dark cycles (14 h/10 h) at 28°C ± 0.5°C. Larvae older than 5 dpf (and until 27 dpf) were kept in nursery tanks in 500–600 mL of 50% 0.3× Danieau's solution and 50% system water, shifting gradually to 100% system water. Starting from 7 dpf, larvae were fed with granular food (SDS100 to SDS400) and from 11 dpf with live feed ( *Artemia salina* ).

### 2.2 NAXD and NAXE Protein Sequence Alignments

Multiple sequence alignments were performed with the EMBL‐EBI Clustal Omega tool using default settings and with the indicated protein sequences retrieved from the NIH NCBI protein database. The Jalview software (version 2.11.2.3) was used to edit and annotate the alignment.

### 2.3 Expression, Purification, and Enzymatic Activity Assay of Recombinant Zebrafish Naxd

A pET‐28a expression vector containing the XM_005167464.4 sequence for expression of zebrafish Naxd isoform X7 with an N‐terminal hexahistidine tag was obtained from GenScript. Overexpression in *Escherichia coli* and purification of the recombinant zebrafish His‐Naxd protein were performed as described for other recombinant proteins in [24].

The NAD(P)HX dehydratase activity of recombinant purified zebrafish Naxd was assayed by spectrophotometric monitoring of S‐NADHX consumption at 290 nm as described in [2]; the S‐NADHX substrate was prepared as described in [24]. S‐NADHX was added at a final concentration of 50 μΜ in the assay mixture, and the reaction was started by enzyme addition at a final concentration of 10 μg/mL. Human recombinant NAXD (10 μg/mL), prepared as previously described [2], was used in positive control assays.

### 2.4 Generation of CRISPR/Cas9 Zebrafish Mutants

Target sequences for sgRNA design in the *naxe* (NM_001002618.1) and *naxd* (NM_001328150.1) genes were selected using the online tool CHOPCHOP [31], and the sgRNA sequences used in this study were the following: Zf_naxe_exon_5: 5′‐CATCTGTATTGTACCCGAAGCGG‐3′; Zf_naxd_exon_5: 5′‐CACAGUGUGGUUGUGGGACC‐3′. Custom‐made sgRNAs were purchased from Sigma‐Aldrich, and stock solutions were prepared by resuspension in distilled water (200 ng/μL). SgRNAs targeting the *naxd* or *naxd* genes (200 ng in 1 μL) as well as the *slc45a* gene (100 ng in 0.5 μL) were microinjected together with Cas9 NLS protein from *Saccharomyces pyogenes* (160 ng in 1 μL, NEB) into one‐cell embryos as described previously [29]. The injected larvae were pre‐selected based on the appearance of an albino phenotype (due to *slc45a* sgRNA) and raised to adulthood. PCR analysis of DNA extracted from whole larvae or adult fin biopsies and stable mutant line generation were performed as described previously [29]. For the identification of indel mutations in the *naxe* and *naxd* genes, PCR amplicons from F1 fish (see Table S1 for primer sequences) were purified using the QIAquick PCR Purification Kit (Qiagen, catalog no. 28104), sequenced (Eurofins), and compared to the corresponding WT sibling sequences.

### 2.5 Early Genotyping in Live Zebrafish Larvae

Live genotyping was performed on 4 dpf larvae as described previously [32], except that larvae were incubated for 45 min with 25 μg/mL proteinase K at 37°C and with shaking at 230 rpm. The DNA lysate (8 μL) was added to a PCR reaction (total volume 16 μL) containing forward and reverse primers (1 μΜ each, Table S1) for the gene of interest and 7.5 μL DreamTaq Green PCR Master Mix (Thermo Fisher Scientific, catalog no. K1081). The PCR program followed the manufacturer's instructions, and amplicons were visualized on 2.5% agarose gels.

### 2.6 Survival Assessment

To evaluate the survival of larvae over time, WT and homozygous mutant larvae at 6 dpf were transferred to nursery tanks for daily observation, and any dead or severely cachectic larvae were recorded and removed. Survival curves were based on at least three independent survival assessments, each with 30–50 larvae per genotype.

### 2.7 Lipopolysaccharides (LPS) and Nicotinic Acid Treatment

For LPS treatment, 25 larvae at 4 dpf were transferred into each well of six‐well plates containing 2 mL 0.3× Danieau's solution with 25 μg/mL LPS from *Salmonella typhosa* (Sigma‐Aldrich, catalog no. L7895) and incubated for 24 h at 28°C. For nicotinic acid treatment, 7 dpf larvae were incubated in 100 μM nicotinic acid (Sigma‐Aldrich, catalog no. N4126) dissolved in the system water. The nicotinic acid medium was renewed daily until the endpoint of each experiment. The final concentrations for both LPS and nicotinic acid treatment were chosen after performing dose–response experiments and evaluating potential toxicity for both reagents based on daily observation for dysmorphia or lethality. For LPS, a concentration range of 10–30 μg/mL was tested, and concentrations greater than 25 μg/mL were found to be toxic for both WT and mutant larvae. For nicotinic acid, a concentration range of 10–500 μΜ was tested, and no toxicity was observed; therefore, a working concentration 15 times higher than the one used in a previous study [33] was selected for nicotinic acid treatments.

### 2.8 Larvae Head Dissection and RNA Extraction for qPCR and Transcriptomics Analyses

Larvae were euthanized by hypothermia, and RNA was extracted from 30 larvae at 5 dpf, 10 larvae at 10 dpf, heads (dissected with a surgical scalpel) of 45 larvae at 5 dpf, or adult zebrafish organs using TRIzol reagent and bead‐beating as previously described [29]. RNA concentration was determined by measuring A260 (NanoDrop 2000). For transcriptomics analysis, 2 μg of total RNA from either heads or whole larvae was treated with RNase‐free DNase I (Sigma‐Aldrich, catalog no. 69182) following manufacturer's recommendations, the RNA quality was evaluated using a 2100 Bioanalyzer (Agilent Technologies), and RNA preparations with an RNA integrity number (RIN) of 8.30–9.40 were used for RNA‐seq analysis by BGI Tech Solutions (Hong Kong), who also provided the Dr. Tom program (https://biosys.bgi.com) for data analysis. Each RNAseq experiment was performed with five biological replicates per genotype, where each replicate sample was derived from 10 larvae at 10 dpf or 45 larval heads at 5 dpf.

Total RNA reverse transcription and qPCR reactions were performed as described in [29] (qPCR primer sequences are given in Table S1). Each experiment was performed in three to five biological replicates, with technical duplicates per biological replicate. Relative gene expression levels were calculated using the 2−^ΔΔC*t*^ method [34], with *actb1* and/or *rpl13* as reference genes.

### 2.9 Locomotor Behavior Assays

Twenty‐four hours prior to recording, larvae were placed in 24‐well plates containing 1 mL of Danieau's medium per well, and the swimming behavior was recorded under normal light conditions (50% of total light intensity) for 5, 7, 10, and 13 dpf larvae with the DanioVision system (Noldus Information Technology) as described in Supporting Information S1.

### 2.10 Neutral Red Staining and TUNEL Assay

Lysosomal compartment and apoptotic cell detection in zebrafish larvae were performed by neutral red staining and TUNEL assay, respectively, as described in Supporting Information S1.

### 2.11 Targeted NAD(P)(H)(X) Measurements by LC–MS

Metabolites were extracted from batches of larvae (35 larvae per batch at 5 dpf, 10 larvae per batch at 10 and 13 dpf) euthanized by hypothermia, and NAD(P)(H)(X) metabolites were measured by LC–MS, as described in Supporting Information S1. Manual peak integration was performed using the software indicated in Table S2. Raw LC–MS peak areas, normalized peak areas, and quantified values are provided in Table S3.

### 2.12 Statistical Analyses

Statistical analyses were conducted as detailed in the figure legends using GraphPad Prism (version 9.0). *T*‐tests or ANOVA with multiple comparisons were performed. For the latter, we did not apply corrections for multiple testing, as only a limited number of predefined biological comparisons were considered (and therefore the likelihood of inflated false positives was low).

## 3 Results

### 3.1 Evolutionary Conservation and Expression of the Zebrafish Naxd and Naxe Proteins

Depending on the transcription initiation site and alternative splicing, the human *NAXD* gene, encoding the NAD(P)HX dehydratase enzyme, can produce multiple transcripts encoding three protein products with different subcellular localizations (cytosolic, mitochondrial, and ER) [28]. The zebrafish genome contains only one orthologous gene candidate of human *NAXD*, and for this gene (*naxd*), nine transcripts and corresponding proteins can be found in the NCBI Gene database. Two RefSeq proteins, ATP‐dependent (S)‐NAD(P)H‐hydrate dehydratase isoform 1 (NP_001315079.1) and isoform 2 (NP_001103590.1), are maintained independently of annotated genomes, whereas seven proteins (isoforms X1–X7) are associated with the GRCz11 primary assembly. For further analysis, we focused on the two main isoforms and the X7 isoform (XP_005167521.1), which shares the highest sequence identity (68% based on the NCBI BLASTP tool) with a major human isoform (NP_001229811.1) and, like the latter, is predicted to localize to the mitochondria based on TargetP 2.0 [35].

**FIGURE 1:**
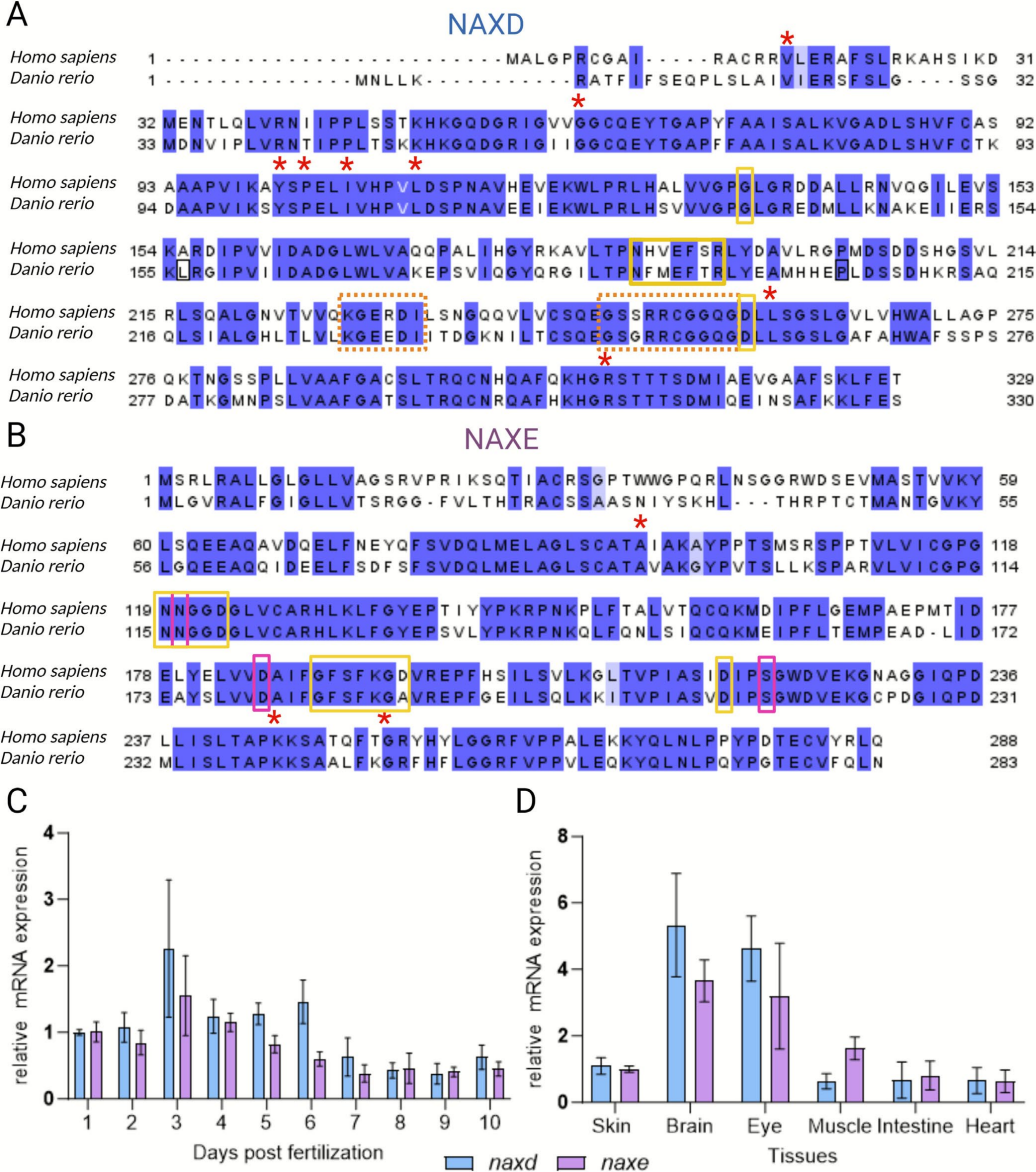
Conservation and expression of zebrafish *naxd* and *naxe*. Alignment of human and zebrafish NAXD (A) and NAXE (B) protein sequences (human NAXD, NP_001229811.1; zebrafish Naxd, XP_005167521.1; human NAXE, NP_658985.2; zebrafish Naxe, NP_001002618.1). Strictly conserved residues and similar residues are highlighted in dark blue and light blue, respectively. Red asterisks indicate residues for which point mutations are known to cause NAD(P)HX repair deficiency in humans [3]. NAD(P)HX binding sites, ATP binding domains (NAXD), and potassium binding sites (NAXE) are highlighted by yellow, dashed orange, and pink boxes, respectively (based on UniProt feature annotation). mRNA expression levels of zebrafish *naxd* (blue) and *naxe* (pink) during the first 10 days post‐fertilization (relative to expression levels at 1dpf) (C) and in adult tissues (relative to expression levels in the skin) (D) measured by qPCR. *Elfa* (C) and *rpl13* (D) were used as reference genes, respectively. Data are shown as means ± SDs from three to five (C) and six (D) biological replicates.

A multiple sequence alignment of the selected zebrafish sequences and the human mitochondrial and ER‐targeted (NP_001229810.1) NAXD forms (both containing the conserved sequence that is also expressed as a cytosolic form of the protein) is shown in Figure S1A. Zebrafish isoforms 1 and 2 exhibit longer N‐terminal extensions that lack conservation with the human proteins and, according to in silico predictions using TargetP 2.0, do not seem to include subcellular targeting peptides. Nevertheless, all zebrafish isoforms show a core sequence that is highly conserved with the human sequences and contains the functional domains and residues important for NAXD activity (Figure S1A and Figure 1A). Furthermore, using purified recombinant enzymes and a spectrophotometric assay to monitor S‐NADHX consumption, we could demonstrate functional conservation between the human and zebrafish NAXD proteins at the enzyme activity level (Figure S1B,C).

For the NAD(P)HX epimerase encoded by the *NAXE* gene (formerly known as *AIBP* or *APOA1BP*), two homologs of the human protein can be found in zebrafish: Naxe, also known as Aibp1 or Apoa1bp1, sharing 68% amino acid sequence identity with human NAXE (Figure 1B), and Yjefn3, also known as Apoa1bp2 or Aibp2, sharing 51% sequence identity with human NAXE (zebrafish Aibp1 and Aibp2 share 49% sequence identity). Yjefn3 is a closer homolog to the human YJEFN3 protein (63% sequence identity), strongly suggesting that Naxe is indeed the zebrafish NAD(P)HX epimerase. It is important to point out that a previous study examined the effects of *aibp* knockdown in zebrafish, suggesting a role for this gene in cholesterol efflux and the control of angiogenesis [36]; in that previous work, it was the *yjefn3* (or *aibp2*) gene that was targeted for silencing with morpholinos, not the *naxe* (or *aibp1*) gene. For both the human *NAXE* and zebrafish *naxe* genes, a single transcript encoding a single protein is found in databases (NP_658985.2 for human and NP_001002618.1 for zebrafish) (Figure 1B).

Based on the transcriptome data in the zfRegeneration database [37], it appears that the zebrafish *naxe* gene is overall expressed at lower levels than the *naxd* gene throughout tissues and developmental stages. We examined the expression levels of both genes by qPCR during embryonic and larval development (1–10 dpf) and in various adult organs (Figure 1C,D). We found that *naxe* and *naxd* are expressed in all examined developmental stages and adult organs, with the highest expression in neuronal tissues (brain and eye). This suggests that the *naxd* and *naxe* genes play an important role, particularly in the neuronal system, which would be in agreement with the predominantly neurological manifestations in the associated human diseases [3].

### 3.2 Generation of Stable *Naxd* and *Naxe* Knockout Lines Using CRISPR/Cas9

**FIGURE 2:**
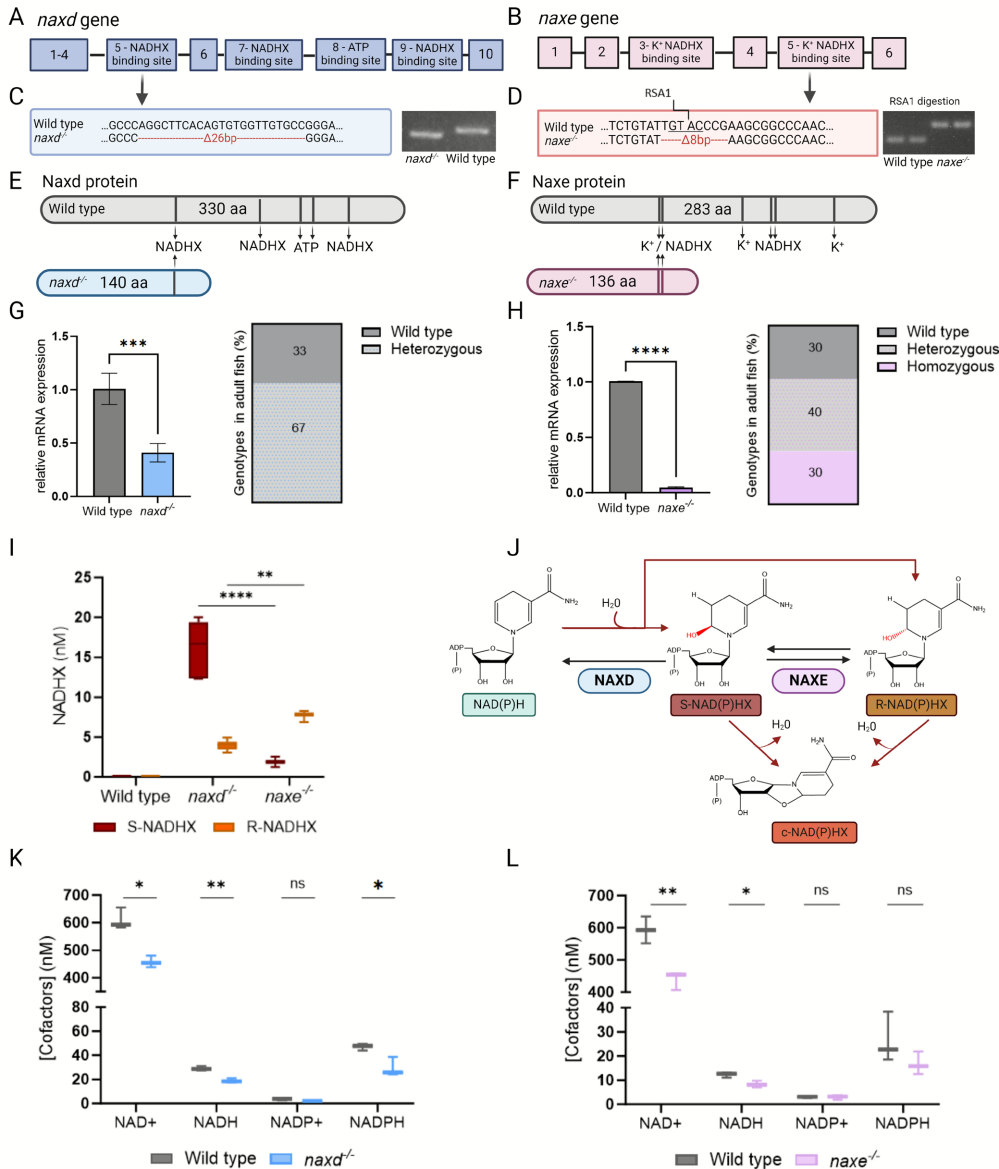
Generation and functional validation of *naxd* ^*−/−*^ and *naxe* ^*−/−*^ knockout lines. (A, B) Schematic representation of the zebrafish *naxd* and *naxe* genes, with boxes representing exons. (C, D) Deletion mutations at the CRISPR target sites of *naxd* and *naxe*, identified in the mutant lines by sequencing at both genomic DNA and cDNA levels. Agarose gels show PCR amplicons obtained from whole larvae genomic DNA for genotyping purposes. For *naxe* ^*−/−*^ identification, amplicons were treated with RsaI enzyme (whose restriction site is lost in the mutant line), prior to gel electrophoresis. (E, F) Schematic representation of WT and predicted truncated Naxd and Naxe proteins. (G, H) *Naxd* and *naxe* transcript levels in homozygous mutant larvae relative to WT siblings. qPCR was performed with RNA extracted from 5 dpf whole larvae, and *actb1* was used as a reference gene. Data are means ± SDs from four biological replicates, each replicate consisting of a batch of 30 larvae. Percentages of WT, heterozygous, and homozygous mutant fish that reached adulthood are visualized in a vertical slice chart. (I) S‐NADHX (red) and R‐NADHX (orange) concentrations in extracts of 10 dpf larvae. Data are means ± SDs from three to six biological replicates, each replicate consisting of a batch of 10 larvae. (J) Schematic representation of the NAD(P)HX repair system. NAD(P)H can be hydrated, either spontaneously or by enzymatic side reactions, to form two epimers, S‐NAD(P)HX or R‐NAD(P)HX. The latter can further react to irreversibly form cyclic NAD(P)HX. NAXE catalyzes the interconversion between the S and R forms of NAD(P)HX. NAXD stereospecifically dehydrates S‐NAD(P)HX back to NAD(P)H. (K, L) NAD(H) and NADP(H) concentrations in extracts of 10 dpf larvae (raw data are provided in Table S3, experiment FERZF). Data shown are means ± SDs from three biological replicates, each replicate consisting of a batch of 10 larvae. Statistical significance was determined with unpaired t‐tests (*p ≤ 0.05, **p ≤ 0.01, ***p ≤ 0.001, and ****p ≤ 0.0001).

To study the in vivo function of the Naxd and Naxe proteins, stable mutant zebrafish lines were generated using CRISPR/Cas9 technology. For both genes, gRNAs were designed to target exon 5, which contains S‐NADHX binding residues (Naxd and Naxe) and K^+^ binding residues in Naxe (Figure 2A,B). The *naxd* mutant line (institutional code *naxd* ^lux6^, hereafter referred to as *naxd* ^*−/*−^) carries a 26‐bp deletion in exon 5, while the *naxe* mutant line (institutional code *naxe* ^lux8^, hereafter referred to as *naxe* ^*−/−*^) carries an 8‐bp deletion in exon 5. For the *naxd* ^*−/*−^ line, the 26 bp deletion allele could be discriminated from the WT allele after PCR amplification. The 8‐bp deletion in the *naxe* ^*−/−*^ line disrupts the recognition site for the RsaI restriction enzyme; thus, the PCR product was further digested with RsaI before gel electrophoresis analysis. Both deletions were confirmed at the genomic DNA and cDNA levels by sequencing (Figure 2C,D), and in silico analyses predict early stop codons resulting in truncated Naxd and Naxe proteins of 140 aa and 136 aa, respectively (Figure 2E,F). The Naxd truncation product lacks two S‐NADHX and both ATP binding domains, and the truncated Naxe protein lacks two NADHX binding sites and two potassium binding residues. It is therefore likely that the truncated proteins, if expressed, have a complete loss of function, and that both the *naxd* ^*−/−*^ and *naxe* ^*−/−*^ lines correspond to full knockout lines.

To examine the viability of homozygous *naxd* and *naxe* mutants, heterozygous adults were crossed, offspring (F2) were raised to adulthood, and at 3 months, adult fish were genotyped by fin biopsy. Approximately 30% of the F2 generation were homozygous for the *naxe* mutation, suggesting that *naxe* deficiency is viable in zebrafish under standard conditions. In contrast, the *naxd* F2 population consisted of 67% heterozygous and 33% WT fish, showing that complete loss of Naxd function is lethal (Figure 2G,H). As no adult *naxd* ^*−/−*^ fish could be obtained, live genotyping was performed in the offspring (4–5 dpf larvae) of *naxd* ^+/−^ heterozygous crosses for identification of *naxd* ^*−/−*^ larvae. Analyses by qPCR in 5 dpf *naxd* ^*−/−*^ and *naxe* ^*−/−*^ larvae showed an approximately 60% and 90% decrease in corresponding transcript levels compared to WT siblings, indicating that mutated transcripts undergo nonsense‐mediated mRNA decay (Figure 2G,H).

Given that commercially available antibodies against human NAXD and NAXE proteins did, in our western blot trials, not cross‐react with the zebrafish homologous proteins, we quantified the substrates (S‐NADHX and R‐NADHX) of the deleted enzymes by LC–MS for functional validation of our knockout lines. As expected, S‐NADHX, the direct substrate of NAXD, accumulated to a greater extent than R‐NADHX in *naxd* ^*−/−*^ larvae, whereas the opposite was observed in *naxe* ^*−/−*^ larvae (all at 10 dpf) (Figure 2I,J). In the latter, Naxd can still convert S‐NADHX back to the normal cofactor (Figure 2J); accordingly, total NADHX accumulated at twofold higher levels in *naxd* ^*−/−*^ compared to *naxe* ^*−/−*^ larvae. As for cell extracts from healthy human subjects, S‐NADHX and R‐NADHX were not detected in extracts from WT zebrafish larvae. Targeted LC–MS analyses also revealed that both mutant lines have decreased levels of the healthy NAD^+^ and NADH cofactors compared to WT siblings, while NADPH levels were only decreased in *naxd* ^−/−^ larvae; NADP^+^ was not changed between WT and mutant larvae (Figure 2K,L). These results show that deficiencies in Naxd and Naxe not only lead to the accumulation of NADHX but also affect the levels of the physiological nicotinamide cofactors. Although, the damaged NADHX cofactors accumulated to a greater extent in *naxd* ^−/−^ than in *naxe* ^*−/−*^ larvae, it was surprising to see such contrasting phenotypes (lethal vs. viable) between the two mutant lines.

### 3.3 *Naxd* Deficiency Leads to Premature Death in Zebrafish

**FIGURE 3:**
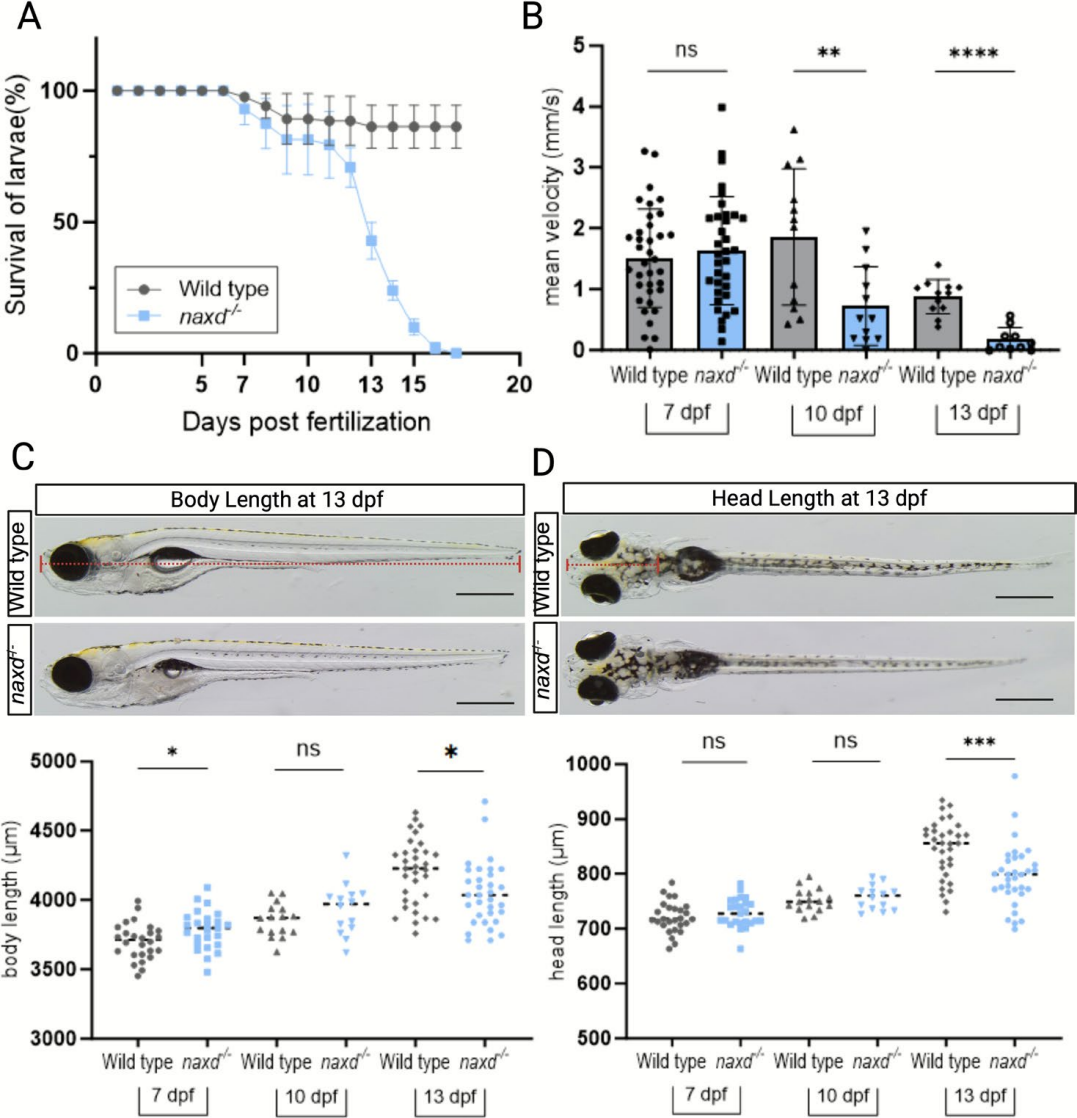
Survival, locomotion, and size analyses in WT and *naxd* ^*−/−*^ larvae. (A) Survival analysis over 20 days. Data are means ± SDs of three independent survival experiments starting with *n* = 20–50 larvae per genotype. (B) Locomotion behavior at the indicated time points. Scatter bar plots represent means ± SDs of the mean velocity calculated after continuous tracking for 30 min in light conditions (50% light intensity); each dot represents one larva (7 dpf, *n* = 36 per genotype; 10 and 13 dpf, *n* = 10–12 per genotype). (C, D) Representative images of WT and *naxd* ^*−/−*^ larvae at 13 dpf in lateral and dorsal view. The scale bars represent 500 μm. Body length (red dotted line in (C)) and head length (red dotted line in (D)) were calculated with the Olympus cellSens Entry software (version 4.1.1). Bottom panels show individual values for the indicated time points, each dot representing one larva (*n* = 25–40 per genotype and time point); horizontal lines correspond to mean values. Statistical significance was determined with unpaired t‐tests (*p ≤ 0.05, **p ≤ 0.01, and ***p ≤ 0.001; ns, not significant).

As *naxd* ^*−/−*^ fish do not reach adulthood, we performed survival studies over 20 days. The survival of *naxd* ^*−/−*^ larvae did not exceed 17 dpf, with a drop to 50% viable larvae between Days 13 and 14 (Figure 3A). On the contrary, the population of WT siblings remained stable after a modest drop in survival between 7 and 11 dpf that occurs typically in this type of survival experiment (Figure 3A).

To start elucidating the reasons for premature death in *naxd* ^*−/−*^ larvae, we analyzed the swimming behavior and the size of developing larvae in a longitudinal manner, performing measurements at an early time point before feeding (day 7), an intermediate time point after feeding where the population is still relatively stable (day 10), and a late time point at which specifically the population of homozygous mutant larvae decreases sharply (day 13). While no differences in swimming velocity were observed between WT and mutant larvae at 7 dpf, *naxd* ^*−/−*^ larvae exhibited a significantly reduced swimming velocity at the later time points, with a 40% and 80% decrease at 10 and 13 dpf, respectively (Figure 3B). For whole body size and head length, we found no notable changes between WT and *naxd* ^−/−^ larvae at 7 and 10 dpf, whereas an about 5% decrease was measured for both parameters in the mutant larvae at 13 dpf (Figure 3C,D).

Naxd (and Naxe) proteins are targeted to several subcellular localisations [28], but are enriched in mitochondria, where NADH production is central for this organelle's energetic role. In *NAXD* patient fibroblasts, decreased levels of certain OXPHOS complex subunits and reduced viability in galactose‐containing media pointed toward impaired mitochondrial function [2]. Although in‐depth analyses of mitochondrial function were beyond the scope of this study, western blotting experiments (performed as described in Supporting Information S1) revealed significantly increased levels of the complex I subunit NDUFB8 and of the complex V subunit ATP5A in 13 dpf *naxd* ^−/−^ larvae (no differences were detected at 10 dpf) (Figure S2), indicating increasing oxidative, metabolic and/or other types of stress levels in developing *naxd* ^−/−^ larvae that may trigger a compensatory upregulation of certain OXPHOS complex subunits.

In summary, we could not detect gross phenotypic changes in *naxd* ^−/−^ larvae during the first week of development. However, notable phenotypic manifestations began to emerge around 10 dpf, progressively intensifying as survival rates declined. Further work is needed to understand whether this delayed onset of phenotypic alterations is due to maternal RNA compensatory effects, a gradual accumulation of NAD(P)HX and/or decline in NAD(P) cofactors beyond critical thresholds, increasing energy demand and complexity of the developing larvae, or a combination of those and possibly other factors.

### 3.4 *Naxd* ^−/−^ Larvae Exhibit Immune Dysregulation

**FIGURE 4:**
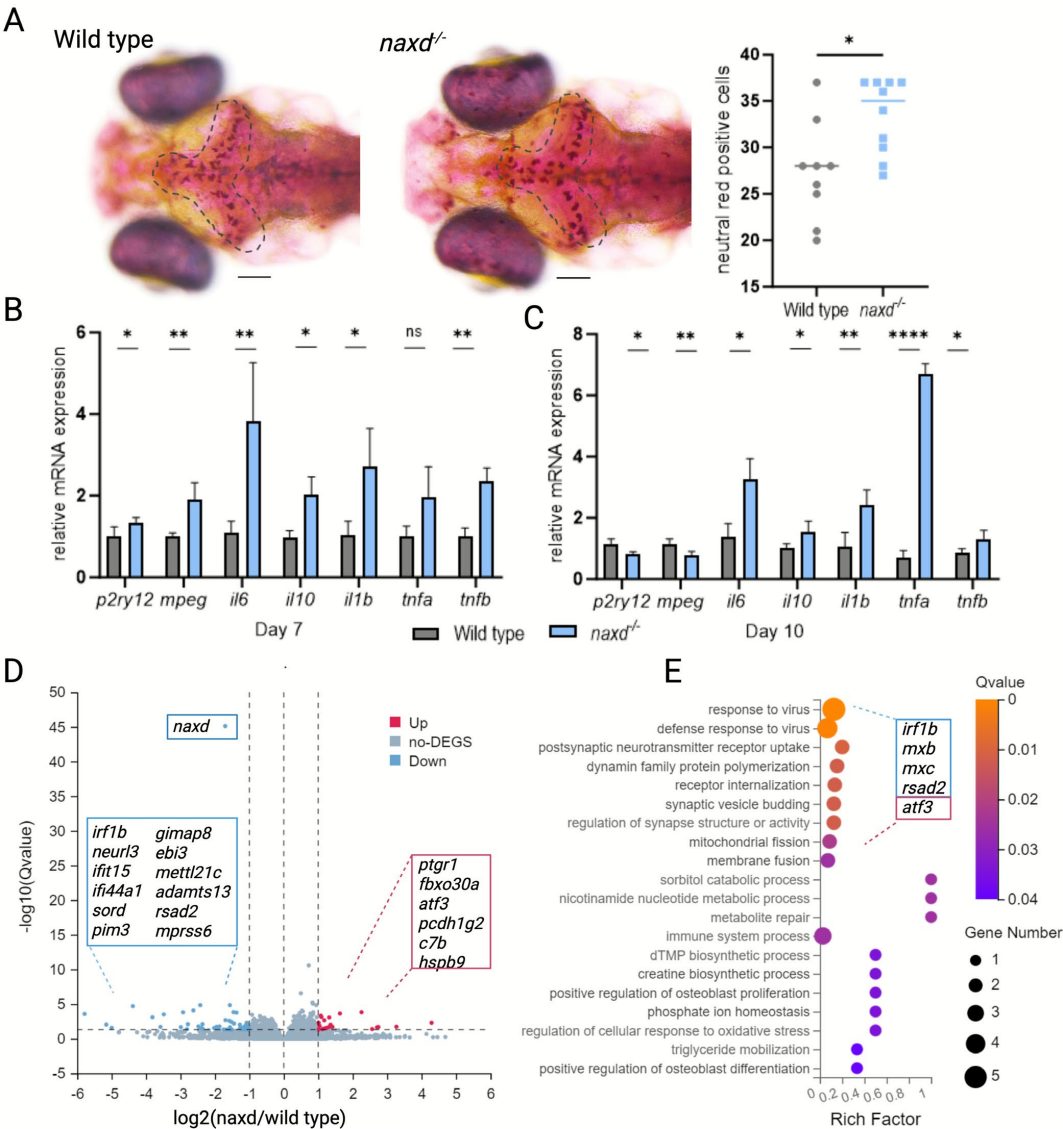
Immune system alterations in *naxd* ^*−/−*^ larvae. (A) Neutral red stain (5 μg/mL for 24 h) of WT and *naxd* ^*−/−*^ larvae at 7 dpf. Stained (microglia/phagocytic) cells were quantified manually with ImageJ. The area used for phagocytic cell number determination is indicated by a dotted line. The scale bars represent 100 μm. Data are shown as individual values, each dot representing one larva (*n* = 9–10 per genotype); horizontal lines represent the means. (B, C) mRNA expression levels of microglia/macrophage markers and cytokines in 7 and 10 dpf *naxd* ^−/−^ larvae relative to WT siblings, based on qPCR analysis on RNA extracted from whole larvae. *actb1* was used as a reference gene. Data are means ± SDs from four to five biological replicates, each replicate consisting of a batch of 30 or 10 larvae at 7 and 10 dpf, respectively. For panels (A–C), statistical significance was determined using unpaired *t*‐tests (*p ≤ 0.05, **p ≤ 0.01, and ****p ≤ 0.0001). (D) Volcano plot highlighting DEGs (*naxd* ^*−/−*^ vs. WT) with FC ≥ 1 and *Q* value ≤ 0.05. Significantly upregulated and downregulated genes are shown as red and blue dots, respectively; gray dots represent genes with nonsignificantly changed expression. (E) Rich factor plot of the GOp enrichment analysis performed on the DEGs. The genes in the blue box correspond to significantly downregulated genes assigned to the viral response pathway, and the gene in the pink box corresponds to a significantly upregulated gene in the same pathway. The *mxb* and *mxc* genes are also attributed to the seven subsequent pathways listed on the *y*‐axis.

Given that disease manifestation in NAXD deficiency depends on inflammatory triggers and that NAXD patients show inflammatory phenotypes, we wanted to explore if inflammatory markers could also be detected in our *naxd* ^*−/−*^ larvae, which spontaneously developed an early lethal phenotype. Neutral red staining indicated an increase in microglia cell numbers or phagocytic activity in *naxd* ^*−/−*^ larvae at 7 dpf (Figure 4A). This observation was further supported by increased expression levels of the *p2ry12* and *mpeg* microglia and macrophage markers in 7 dpf *naxd* ^*−/−*^ larvae based on qPCR analysis in whole larvae (Figure 4B). Interestingly, at a later time point (10 dpf), after the onset of more pronounced phenotypes in *naxd* ^*−/−*^ larvae, the *p2ry12* and *mpeg* transcript levels were decreased in the *naxd* ^*−/−*^ versus WT larvae (Figure 4C), indicating a reversal of the early, possibly compensatory, increase in macrophage function and/or population. Due to decreased penetrance of the staining reagent in 10 dpf larvae, neutral red staining for phagocytic cells could not be performed at this later time point.

Transcript levels of the cytokines *il6, il10, il1b*, and *tnfb* were significantly upregulated in *naxd* ^*−/−*^ larvae compared to WT siblings at 7 dpf (Figure 4B). Upregulation of the cytokine transcript levels was maintained in the mutant larvae at 10 dpf, with *tnfa* reaching a statistically significant and more than sevenfold change (Figure 4C). Increased expression of these pro‐inflammatory cytokines (*il6, il1b, tnfb, and tnfa*) and the anti‐inflammatory cytokine *il10* indicates a dysregulated immune response of *naxd* ^*−/−*^ larvae, with a potential shift toward an inflammatory state.

Next, to examine gene expression changes with an unbiased approach, we performed bulk RNA sequencing in whole *naxd* ^*−/−*^ and WT larvae at 10 dpf. Out of 32.324 detected genes, only 74 were differentially expressed between *naxd* ^*−/−*^ and WT siblings with a log2FC ≥ 1 and *Q* value ≤ 0.05 (Table S4). Among those DEGs, 53 genes were downregulated, and 21 were upregulated in *naxd* ^*−/−*^ versus WT samples, and as expected, the most significantly downregulated gene in *naxd* ^*−/−*^ larvae was the *naxd* gene. After removing the genes of unknown function, 33 downregulated and 15 upregulated genes remained for further analysis.

The 12 most significantly downregulated genes (*Q* value ≤ 0.001) include 8 genes that are induced or stimulated by interferons or induce their production: the interferon regulatory factor *irf1b*, *neurl3*, which regulates the interferon regulatory factor 7 and augments the host antiviral response [38], *ebi3* and *il‐27*, that participate in pathogen immune response through IFN stimulation [39], and the antiviral response IFN‐stimulated genes *ifit15, ifi44a1*, *rsad2*, and *viperin* [40, 41, 42] (Figure 4D). The remaining highly significantly downregulated genes are the serine/threonine kinase *pim3*, which is a positive regulator of T‐cell cytokine production [43], *gimap8*, which belongs to the immuno‐associated nucleotide‐binding protein family [44], and finally, genes associated with other diseases such as juvenile amyotrophic lateral sclerosis and neuropathy (*sord*), sarcopenia and muscle dystrophies (*mettl21c*), thrombotic thrombocytopenic purpura (*adamts13*), and microcytic anemia (t*mprss6*) [45, 46, 47, 48, 49, 50] (Figure 4D).

The most significantly upregulated genes (*Q* value ≤ 0.003) are functionally more diverse and include immune and neuronal function‐related genes as well as stress response genes. Immune‐related genes are the prostaglandin reductase 1 (*ptgr1*), which is a rate limiting NAD(P)H‐dependent enzyme of the inactivation pathway of prostaglandins [51] and plays key roles in both cardiovascular and immune systems [52, 53], and the complement component 7 (*c7b*), which not only partakes in the pathogen response [54] but also in recognition and removal of apoptotic and necrotic cells [55] (Figure 4D). Upregulated genes that are attributed to neuronal function are protocadherin 1 gamma (*pcdh1g2*), which is required for synaptic development and affects the survival of cortical neurons [56, 57], and the F‐box protein 30 (*fbxo30a*), which has been connected with neural tube defects [58] (Figure 4D). Finally, stress‐related genes include the activating transcription factor 3 (*atf3*), which is mainly expressed in myeloid cells, including microglia, upon a stress stimulus [59, 60] and the heat shock protein alpha crystallin (*hspb9*), which acts as a chaperone and binds to denaturing proteins, thus protecting the cells from protein aggregate induced toxicity [61, 62]. Of note, *atf3* and *c7b* have been shown to co‐express during regeneration processes of the retina [63].

GOp enrichment analysis performed with the total DEGs revealed the pathogen response as the most significantly affected pathway, and all the genes attributed to it were downregulated except for one (*c7b*). In addition to the abovementioned downregulated genes, this pathway includes the IFN‐stimulated genes, *mxb* and *mxc* (Figure 4E). Taken together, these results strongly point to immune function dysregulation as an early phenotype in *naxd* ^*−/−*^ larvae, and future work should be directed toward understanding its connection to the observed increased stress levels and causal role (or not) in the early lethality of these larvae.

### 3.5 Nicotinic Acid Treatment Increases the Survival of *naxd* ^*−/−*^ Larvae

**FIGURE 5:**
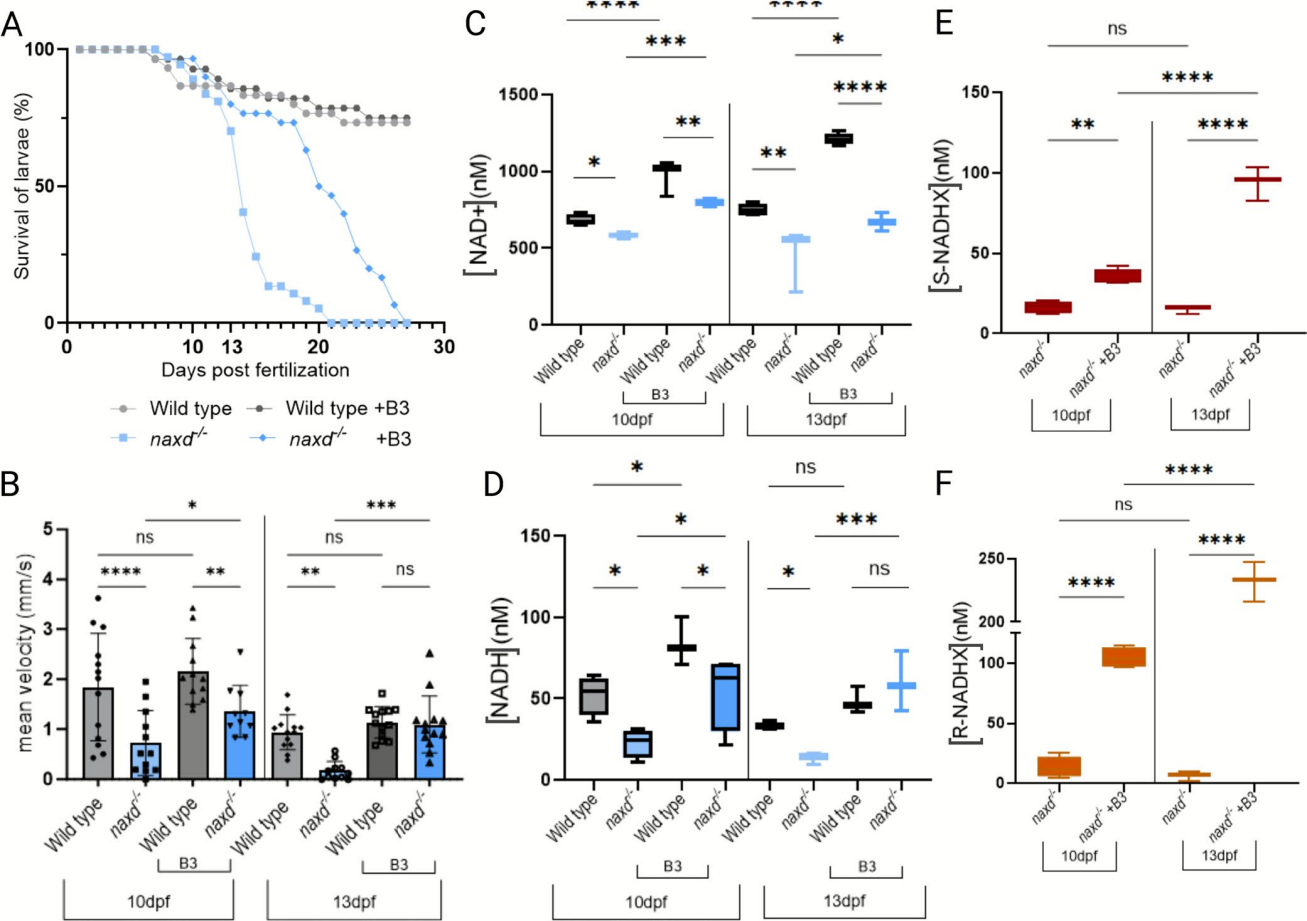
Nicotinic acid supplementation (partially) rescues survival and locomotor defects in *naxd* ^−/−^ larvae. (A) Survival analysis of WT and *naxd* ^*−/−*^ larvae without and with 100 μM nicotinic acid (B3) supplementation over 30 days (*n* = 30–50 larvae per genotype and condition). B3 supplementation was started at 7 dpf, and the water without or with the supplement was refreshed daily. (B) Locomotion behavior of untreated and B3‐supplemented WT and *naxd* ^*−/−*^ larvae determined at 10 and 13 dpf. Scatter bar plots represent means ± SDs of the mean velocity calculated after continuous tracking for 30 min in light conditions (50% light intensity); each dot represents one larva (*n* = 10–12 per genotype and condition). (C–F) NAD(H), S‐ and R‐NADHX concentrations in extracts of 10 or 13 dpf larvae are shown as means ± SDs from three to four biological replicates, each replicate consisting of a batch of 10 whole larvae (raw data are provided in Table S3, experiment DOV33). Statistical significance was determined by one‐way ANOVA with multiple comparisons test (*p ≤ 0.05, **p ≤ 0.01, ***p ≤ 0.001, and ****p ≤ 0.0001; ns, not significant).

Given that high‐dose vitamin B3 (100–500 mg/day) treatment showed beneficial effects in *NAXE* and *NAXD* deficient patients [9, 15], and that supplementation with nicotinic acid (NA), a B3 vitamer, was reported to ameliorate phenotypes in zebrafish models of muscular dystrophies [33, 64], we next tested this treatment in *naxd* ^*−/−*^ larvae. NA supplementation, at a final concentration of 100 μΜ, was initiated at 7 dpf and maintained until the endpoint of the experiment at 28 dpf by daily medium refreshment. Remarkably, NA treatment substantially prolonged the survival of *naxd* ^*−/−*^ larvae compared to the untreated mutant siblings, without affecting the survival of the WT siblings (Figure 5A). Furthermore, the swimming velocity of treated *naxd* ^*−/−*^ larvae improved significantly at 10 dpf and was even fully restored to WT levels by 13 dpf (Figure 5B). However, after 20 dpf, the survival of treated *naxd* ^*−/−*^ larvae started to decrease, and they did not survive past 27 dpf (Figure 5A).

LC–MS analysis revealed that NA treatment significantly and time‐dependently increased NAD^+^ and NADH levels in both WT and *naxd* ^−/−^ larvae (Figure 5C,D). This was, however, accompanied by a concomitant increase in S‐ and R‐NADHX levels in the *naxd* ^*−/−*^ larvae (Figure 5E,F), while these damaged cofactors remained undetectable in NA‐treated WT larvae. This clearly suggests that the beneficial effect of NA treatment is mediated by boosting the normal cofactor levels and that this can transiently overcome the presumably toxic effects of the concomitantly increasing levels of the damaged cofactors in the *naxd* ^*−/−*^ larvae. These findings support the promise that supplementation with NA and potentially other NAD precursors holds for the treatment of the PEBEL disorders and confirm the utility of our *naxd* ^*−/−*^ zebrafish line as an in vivo model for preclinical testing of the efficiency of new therapeutic compound candidates. At the same time, our results signal that caution must be taken with chronic administration of vitamin B3, since, in the long run, the organism may not be able to cope with the NADHX overload that inevitably will accompany the increased levels of healthy cofactor in the absence of a functional NADHX repair system.

### 3.6 Gene Expression Analyses Reveal Immune Dysregulation Also in *naxe* ^*−/−*^ Larvae Despite Absence of Overt Phenotypes

In agreement with previous observations in *Naxe* knockout mice [65, 66, 67], but in stark contrast to the *naxd* ^−/−^ zebrafish larvae, *naxe* ^−/−^ larvae did not exhibit any notable changes in viability or locomotor behavior under standard conditions compared to WT siblings (Figure S3).

Given the neurological similarities between *NAXE* and *NAXD* deficient patients, we nevertheless wanted to investigate molecular differences in the central nervous system between *naxe* ^*−/−*^ and WT larvae. As described in more detail in Supporting Information S1, bulk RNA sequencing of heads from 5 dpf *naxe* ^−/−^ larvae revealed downregulation of many immune‐related genes compared to WT siblings (Figures S4 and S5, Table S5). Similar to *naxd* ^−/−^ larvae, neutral red staining indicated an increased microglial cell number or phagocytic activity in *naxe* ^−/−^ larvae, accompanied by elevated expression of the microglial marker *p2ry12* (Figure S4). No significant difference in apoptotic cell numbers was found in the head region between WT and *naxe* ^−/−^ larvae based on TUNEL staining (Figure S4). Furthermore, qPCR analysis of whole 5 dpf *naxe* ^−/−^ larvae showed reduced basal expression of inflammatory cytokines and a striking absence of cytokine induction following LPS treatment (Figure S6). These results show clear immune system abnormalities also in the *naxe* ^−/−^ larvae despite the absence of apparent neurodevelopmental defects.

## 4 Discussion

PEBEL is a severe neurometabolic disorder that manifests in the first months or years of life and is caused by a deficiency in one of the two enzymes that constitute the NAD(P)HX metabolite repair system (NAXE or NAXD). As this disorder is both rare and lethal, studying the underlying causative molecular mechanisms is challenging. Previous work from our group based on budding yeast and nearly haploid (HAP1) human cell models provided the first insights into the molecular consequences of *NAXE* and *NAXD* deficiencies in living cells [24, 27]. In both cellular models (yeast and HAP1), *NAXE* deficiency led to moderate increases in damaged nicotinamide cofactor levels but no other detectable phenotypes, while *NAXD* deficiency led to more pronounced NADHX accumulation, accompanied by NAD^+^ depletion in the yeast cells, serine depletion in both yeast and human cells, and decreased viability upon prolonged culture or under galactose stress in HAP1 cells.

While *NAXD* deficiency has not been studied in vivo so far, a *Naxe* KO mouse model has been described previously [67, 68]. *Naxe* null mice were viable and fertile and did not present any morphological abnormalities under standard conditions [67]. NAXE has a secondary proposed function as a secreted Apolipoprotein AI‐binding protein (AIBP) [69, 70] and is reported to be involved in cholesterol metabolism, lipid raft formation, angiogenesis, atherogenesis, inflammation, and tumorigenesis [66, 67, 68, 71]. Actually, the first study suggesting a role for NAXE in cholesterol efflux and angiogenesis also included studies on *aibp* morphant zebrafish [36]. As explained earlier, the zebrafish genome contains two AIBP‐homologous genes, *aibp1* and *aibp2*. Based on evolutionary sequence conservation, the zebrafish Aibp1 and Aibp2 proteins have been renamed Naxe and Yjefn3, respectively. Yjefn3 is also conserved in humans, where its function remains unclear. Fang et al. [36] showed that zebrafish Aibp2 (Yjefn3), but not Aibp1 (Naxe), promotes cholesterol efflux from cells and all subsequent experiments supporting a role for Aibp in angiogenesis in zebrafish stem from larvae in which *aibp2* (*yjefn3*), and not *aibp1* (*naxe*, i.e., the gene knocked out in the present study), was knocked down by antisense morpholinos. Conversely, accumulation of NADHX in cell [24] and animal models [72] deficient in NAXE, but with intact YJEFN3, strongly indicates that the latter does not act as an NADHX epimerase. This is further supported by the reported lack of NADHX epimerase activity of mouse Yjefn3 in vitro [28] and by the lack of conservation of residues that are strictly conserved (and involved in NAD(P)HX binding) in NAXE, but not in YJEFN3 (Figure S7). Finally, our bulk RNAseq analyses did not show any upregulation of *yjefn3* expression in the *naxe* ^−/−^ larvae compared to the WT siblings (mean of FKPM values of 21.50 ± 1.00 and 20.37 ± 1.91 in mutant and WT larvae, respectively), also arguing against a potential compensatory effect of the *yjefn3* gene that would explain the weak phenotype of the *naxe* ^−/−^ compared to the *naxd* ^−/−^ mutant.

We used CRISPR/Cas9 editing to generate the first, to our knowledge, zebrafish models of NAXE (AIBP1) and NAXD deficiency. Both *naxe* ^*−/−*^ and *naxd* ^−/−^ zebrafish larvae showed the characteristic accumulation of NADHX and, in agreement with mouse studies [67, 71], *naxe* ^*−/−*^ larvae were viable, developed apparently normally, and were fertile. By contrast, *naxd* ^−/−^ larvae exhibited a severe phenotype, characterized by decreased locomotor behavior and lethality during the first month of life. Total NADHX accumulated to higher levels in *naxd* ^−/−^ larvae compared to *naxe* ^*−/−*^, with S‐NADHX reaching about eight times higher levels in *naxd* ^−/−^ than in *naxe* ^*−/−*^ larvae. Both *naxd* ^−/−^ and *naxe* ^−/−^ larvae exhibited decreased levels of NAD^+^ and NADH, while *naxd* ^*−/−*^ larvae additionally displayed decreased NADPH levels. Given the similar clinical manifestations of *NAXE* and *NAXD* deficient patients and the qualitatively similar alterations in nicotinamide cofactor levels in *naxe* ^−/−^ and *naxd* ^−/−^ zebrafish, the marked difference in phenotypic severity between these mutants (along with the lack of overt phenotype in *Naxe* knockout mice) is unexpected. It could be that S‐NADHX, which accumulated to considerably higher levels in *naxd* ^−/−^ compared to *naxe* ^*−/−*^ larvae, is more toxic than R‐NADHX. Also, given that R‐NADHX to S‐NADHX conversion can proceed spontaneously, R/S‐NADHX levels may never accumulate in *naxe* ^*−/−*^ larvae (under standard conditions) above a critical threshold, due to the presence of functional Naxd, which constantly removes S‐NADHX from the system. As highlighted previously, the NADHX repair enzymes also act on NADPHX derivatives, which are formed under physiological conditions as well [24, 28]. Those derivatives were not measured in our study, but they likely also contribute to the observed phenotype. Given that the disease onset in both PEBEL1 and PEBEL2 is triggered by stress, we can speculate that a complete loss of function of *NAXD*, as is presumably the case in our *naxd* zebrafish model (but not in the described human *NAXD* cases), is not viable, and that a yet to be identified stress trigger is required to reveal a severe phenotype in the *naxe* zebrafish model. Since NAD(P)HX formation increases with temperature [23] and the zebrafish body temperature corresponds to the surrounding water (28°C under our laboratory conditions), which is considerably lower than in mammals, it will be interesting to determine whether heat stress can unmask a more pronounced phenotype in the *naxe* zebrafish model.

We found increased numbers (or phagocytic activity) of microglia and elevated expression of microglial (*p2ry12*) and macrophage (*mpeg*) markers in 7 dpf *naxd* ^−/−^ larvae. In addition, we measured increased expression of the pro‐inflammatory cytokines *tnfa, tnfb, il1b*, and *il6*, as well as of the anti‐inflammatory cytokine *il10* in *naxd* ^−/−^ larvae at 7 and 10 dpf. Combined with decreased locomotor activity in *naxd* ^−/−^ larvae from 10 dpf onwards, these results are reminiscent of the ones reported for a zebrafish model of leukodystrophy, where impaired microglial phagocytosis of apoptotic cells led to increased expression of pro‐inflammatory cytokines and reduced locomotor activity [73]. Single‐cell RNA sequencing of larval brains [74] as well as the use of transgenic reporter lines could allow to further substantiate the hypothesis that Naxd deficiency leads to early microglial dysfunction resulting in neurodevelopmental changes.

Bulk RNA sequencing of whole *naxd* ^−/−^ larvae at 10 dpf revealed a downregulation of interferon‐related genes. Type I and type II interferons constitute a critical first line of defense against pathogens [75] and play key roles in initiating and modulating the antiviral immune response [76]. Interferons are activated via pattern recognition receptors that detect pathogen‐ and damage‐associated molecular patterns (PAMPS and DAMPS), and they act in concert with other pro‐inflammatory cytokines to mount an effective immune response [77]. The observed upregulation of pro‐inflammatory cytokines may therefore also reflect a compensatory mechanism in response to reduced interferon signaling. GOp enrichment analysis further highlighted the pathogen response as the most significantly affected pathway, with all associated genes being downregulated. Interestingly, among the most upregulated transcripts were genes involved in cellular stress responses, as well as *c7b*, a complement component typically activated by necrotic or apoptotic cells [55]. These findings suggest that at 10 dpf, *naxd* ^−/−^ larvae exhibit increased cellular stress, possibly linked to increased levels of apoptosis.

Although the transcriptomic analyses of *naxe* ^−/−^ and *naxd* ^−/−^ larvae are not directly comparable, having been performed in different tissues (heads vs. whole larvae) and at different developmental stages (5 dpf vs. 10 dpf), it is remarkable that, despite their contrasting phenotypes, both mutant lines showed the most significant alterations in immune‐related genes and pathways.

NAD(H) and NADP(H) are among the most abundant cofactors, and their crucial roles in cell physiology, metabolism, signaling, and also ageing and neurodegeneration have been reviewed extensively [78, 79, 80]. Increasing evidence also indicates that NAD plays diverse, pathway‐specific roles in immunometabolism, driven not only by its impact on cellular metabolic state but also through regulation of sirtuin deacetylases and poly(ADP‐ribose) polymerases (PARPs) [81, 82, 83]. Moreover, NADPH is necessary for both the production of reactive oxygen species through the oxidative burst, a crucial immune response against pathogens [84], and for their detoxification [85]. While more work is needed to elucidate the mechanism by which deficiencies in Naxe and Naxd lead to the observed immune dysregulation in our models, our study strongly supports a role of the NAD(P)HX repair system in immune system development and hence for dysregulation of the latter to participate in the pathophysiology of NAD(P)HX repair disorders.

In this line of thought, it is interesting to mention that leukocytes have recently been shown to drive CNS pathology in a mouse model of Leigh syndrome, the most common form of pediatric mitochondrial disease [86]. The NAXD and NAXE enzymes are enriched in the mitochondria [28] and our prior work in NAXD‐deficient cell models [2, 24, 27] as well as the present study in zebrafish indicate that lack of NADHX repair leads to mitochondrial dysfunction. Furthermore, neuroimaging findings in PEBEL patients resemble brain abnormalities typically found in mitochondrial diseases (e.g., cytotoxic edema of the basal ganglia [3]). Taken together, these findings suggest that the NAD(P)HX repair system may represent a critical link between mitochondrial integrity and immune homeostasis, with potential implications for understanding the immunometabolic basis of neurodegeneration in NAD(P)HX repair and other disorders. Notably, a recent case report described disease onset in a child with NAXD deficiency following routine immunizations in the absence of fever [87], further suggesting a role of NADHX repair in immune system development and its potential relevance to immune‐triggered neurodegeneration.

Finally, in agreement with observations in PEBEL patients [9, 10, 15, 16], nicotinic acid treatment ameliorated the phenotype, rescuing locomotor activity and significantly prolonging the survival of *naxd* ^−/−^ larvae. Nicotinic acid supplementation increased the levels of NAD^+^ and NADH while also increasing the levels of NADHX in the *naxd* ^−/−^ larvae, possibly explaining why they ultimately succumb in the presence of treatment as well. These observations strongly suggest that the beneficial effect of vitamin B3 relies on boosting the normal cofactor pools but also call for caution against chronic vitamin B3 administration in PEBEL patients. Accordingly, a recent *NAXE* case report showed an unfavorable outcome upon treatment with high‐dose vitamin B3, despite initial stabilization [5]. For nine other PEBEL1 or PEBEL2 patients reported so far to have been treated with vitamin B3, a good response to therapy and stabilization of symptoms were described [5, 88].

Our zebrafish models of *naxd* and *naxe* deficiency provide in vivo validation of the NAD(P)HX repair system's (essential) role in neurometabolic homeostasis. While both mutants exhibited NADHX accumulation and decreased NAD(H) levels, *naxd* ^−/−^ larvae showed a severe phenotype characterized by progressive locomotor decline, mitochondrial dysfunction, and early lethality. In contrast, *naxe* ^−/−^ larvae were viable and fertile, with more moderate biochemical alterations and no overt developmental phenotype. Despite these differences, both models revealed immune‐related abnormalities, including increased microglial markers and dysregulated cytokine expression, pointing to a shared role of NAD(P)HX repair in immune system development. Notably, nicotinic acid treatment improved survival and motor behavior in *naxd* ^−/−^ larvae by boosting NAD^+^ levels, although NADHX continued to accumulate, suggesting limitations to this approach and the need for cautious clinical translation. These findings underscore the importance of early diagnosis and therapeutic intervention [6], and establish *naxd* and *naxe* zebrafish as valuable in vivo models for dissecting the immunometabolic basis of NAD(P)HX repair disorders and for guiding the development of targeted treatments.

## Author Contributions

Myrto Patraskaki and Carole L. Linster conceived the study. Myrto Patraskaki generated and analyzed most of the presented data. Najmesadat Seyedkatouli performed the size measurements of zebrafish larvae, western blot analyses, and TUNEL assays, and contributed to the metabolomics analyses. Marc O. Warmoes performed metabolite extractions and measurements, Lisa Schlicker performed metabolomics data analysis, and Maria Lorena Cordero‐Maldonado contributed to the generation of mutant zebrafish lines. Ursula Heins‐Marroquin and Maria Lorena Cordero‐Maldonado contributed to experimental design and supervision of the work as well as to preparation of the ethics approval proposal. Myrto Patraskaki and Carole L. Linster mainly interpreted the presented data. Myrto Patraskaki wrote the first draft of this manuscript as part of her PhD thesis. Carole L. Linster is responsible for the final text of this article. All authors contributed to data interpretation and manuscript revision.

## Funding

This work was supported by Fonds National de la Recherche Luxembourg (PRIDE17/12244779/PARK‐QC, PRIDE19/14063202/ACTIVE, C22/BM/17198760/NAXDivo).

## Ethics Statement

The Zebrafish Facility at the Luxembourg Centre for Systems Biomedicine (LCSB) is registered as an authorized breeder, supplier, and user of zebrafish ( *Danio rerio* ) with Grand‐Ducal Decree of January 20, 2016, and January 26, 2023. All activities related to the use of zebrafish were conducted in accordance with the laws, guidelines, and policies outlined in the European Directive 2010/63/EU on the protection of animals used for scientific purposes. The generation and assessment of the *naxd* and *naxe* mutant lines hold the authorization number LUPA 2019/65, and the maintenance and functional characterization of the *naxd* mutant lines were done under approval LUPA 2023/07. In addition, the performance of fin biopsies for genotyping purposes was authorized by LUPA 2017/04 and LUPA 2022/8. Euthanasia by rapid cooling (hypothermia) was performed according to the approved derogations LUPA 2017/03 and LUPA 2022/6.

## Conflicts of Interest

The authors declare no conflicts of interest.

## Supporting information


**Table S1:** List of primers used in this study.

**Table S2:** jimd70149‐sup‐0002‐TableS2.xlsx.

**Table S3:** jimd70149‐sup‐0003‐TableS3.xlsx.

**Table S4:** jimd70149‐sup‐0004‐TableS4.xlsx.

**Table S5:** jimd70149‐sup‐0005‐TableS5.xlsx.

**Figure S1:** jimd70149‐sup‐0006‐FigureS1‐S7.pdf.
**Figure S2:** jimd70149‐sup‐0006‐FigureS1‐S7.pdf.
**Figure S3:** jimd70149‐sup‐0006‐FigureS1‐S7.pdf.
**Figure S4:** jimd70149‐sup‐0006‐FigureS1‐S7.pdf.
**Figure S5:** jimd70149‐sup‐0006‐FigureS1‐S7.pdf.
**Figure S6:** jimd70149‐sup‐0006‐FigureS1‐S7.pdf.
**Figure S7:** jimd70149‐sup‐0006‐FigureS1‐S7.pdf.

**Data S1:** Supporting Information.

## Data Availability

The Supporting Information files contain all relevant data for this study. No data have been submitted to external repositories.
