# Supplementary material for: Unveiling Immune System Perturbations in Early Development Through Zebrafish Models of NADHX Repair Deficiency
Source: J Inherit Metab Dis. 2026 Feb 1;49(2):e70149. doi: 10.1002/jimd.70149 (PMC12861718; doi:10.1002/jimd.70149)
Supplement: Supplementary file 1 — Table S1: List of primers used in this study. [file JIMD-49-0-s004.docx]

**Table S1. List of primers used in this study**

| **Primer name** | **Sequence (5’ to 3’ direction)** | **Purpose** |
| --- | --- | --- |
| naxe_ex5_285bp frw | CATCATGAGTAAACGTTGGCAT | CRISPR efficiency testing |
| naxe_ex5_285bp-rev | TCTTCTGACACTGGATGGACAG | CRISPR efficiency testing |
| zf_naxd_exon 5_new_ frw | GTGATGAGTGAGTGTGACGGTT | CRISPR efficiency testing |
| zf_naxd_exon 5_new_rev | TGTGCCTTTACCACTGCATATT | CRISPR efficiency testing |
| rna_naxe5_frw | CAGGAAACAACGGAGGAGAC | cDNA sequencing |
| rna_naxe5_rev | CTCTGACGGCACCCTTAAAA | cDNA sequencing |
| zf_naxd exon 5 -1 rna frw | GATGCAGCTCCAGTCATCAA | cDNA sequencing |
| zf_naxd exon 5-1 rna rev | TCCTCTGAGTTTTGACCTCTCA | cDNA sequencing |
| NAXE_Frw_109 | TATAGCCTGGTTGTGGACGC | qPCR |
| NAXE_Rev_109 | TAGCAATGGGCACGGTGATT | qPCR |
| zf_naxd qpcr e5/8 fwd | GGAAGGGAGGACATGCTTCT | qPCR |
| zf_naxd qpcr e5/8 rev | GCTGTCTAGAGGCTCGTGA | qPCR |
| P2ry12 frw | CTTCAGGTCGTCGCTGTTTA | qPCR |
| P2ry12 rev | AGTGCGTTTCCCTGTTGAT | qPCR |
| irf8 frw | GACCTCTCAATGCTGCTGTTGTTC | qPCR |
| irf8 rev | CGCTCATTCTTAATGCCGTCAATGG | qPCR |
| Mpeg 1.1 frw | GGGTTCAAGTCCGTAACCATCTGTAC | qPCR |
| Mpeg 1.1 rev | CTTCTTGCACCAATGTGGCTCC | qPCR |
| Csf1ra frw | CCTGATCCGCAACGTTCATCCT | qPCR |
| Csf1ra rev | GCTTTGGGCAGCATTCTTGAGG | qPCR |
| IL1β frw | TGGACTTCGCAGCACAAAATG | qPCR |
| IL1β rev | GTTCACTTCACGCTCTTGGATG | qPCR |
| il-10 frw | CTTTAAAGCACTCCACAACCCCAA | qPCR |
| il-10 rev | CTTGCATTTCACCATATCCCGCTT | qPCR |
| tnfa frw | GGTGATGGTGTCTAGGAGGAA | qPCR |
| tnfa rev | GGTCTTATGGAGCGTGAAGC | qPCR |
| tnfb frw | TTGAAGATGTTGAAGGAGATG | qPCR |
| tnfb rev | CAAGGTAAATGGTGCTGTAGG | qPCR |
| iglas1.1_zf_frw | AATGACTGCTGGTGTGAGGA | qPCR |
| iglas1.1_zf_rev | ACTTCTCTGAGCCTTGACGG | qPCR |
| hbbe1.2_zf_frw | CGAGAAGGCCACCATTCAAG | qPCR |
| hbbe1.2_zf_rev | GAGCACAGTTTTACCGTGGG | qPCR |
| mhc1_zf_frw | ATGGGGTACTTCGACAGCAA | qPCR |
| mhc1_zf_rev | TTTGGTTGAAGCGCTCCATC | qPCR |
| mhc2_zf_frw | TAGTCCTGGCTGAGTTTGGG | qPCR |
| mhc2_zf_rev | CAGTTTGATGGCTTTGGGGT | qPCR |
| bactin1 frw | CACTGAGGCTCCCCTGAATCCC | HK gene for qPCR |
| bactin1 rev | CGTACAGAGAGAGCACAGCCTGG | HK gene for qPCR |
| Rpl13α fwd | TCTGGAGGACTGTAAGAGGTATGC | HK gene for qPCR |
| Rpl13α rev | AGACGCACAATCTTGAGAGCAG | HK gene for qPCR |

*HK, Housekeeping gene
