# Supplementary material for: Unveiling Immune System Perturbations in Early Development Through Zebrafish Models of NADHX Repair Deficiency
Source: J Inherit Metab Dis. 2026 Feb 1;49(2):e70149. doi: 10.1002/jimd.70149 (PMC12861718; doi:10.1002/jimd.70149)
Supplement: Supplementary file 6 — Figure S1: jimd70149‐sup‐0006‐FigureS1‐S7.pdf. Figure S2: jimd70149‐sup‐0006‐FigureS1‐S7.pdf. Figure S3: jimd70149‐sup‐0006‐FigureS1‐S7.pdf. Figure S4: jimd70149‐sup‐0006‐FigureS1‐S7.pdf. Figure S5: jimd70149‐sup‐0006‐FigureS1‐S7.pdf. Figure S6: jimd70149‐sup‐0006‐FigureS1‐S7.pdf. Figure S7: jimd70149‐sup‐0006‐FigureS1‐S7.pdf. [file JIMD-49-0-s001.pdf]

Unveiling Immune System Perturbations in Early Development through Zebrafish Models of NADHX Repair Deficiency

Myrto Patraskaki, Najmesadat Seyedkatouli, Lisa Schlicker, Marc O. Warmoes, Maria Lorena Cordero-Maldonado, Ursula Heins-Marroquin, and Carole L. Linster

Supplementary Figures

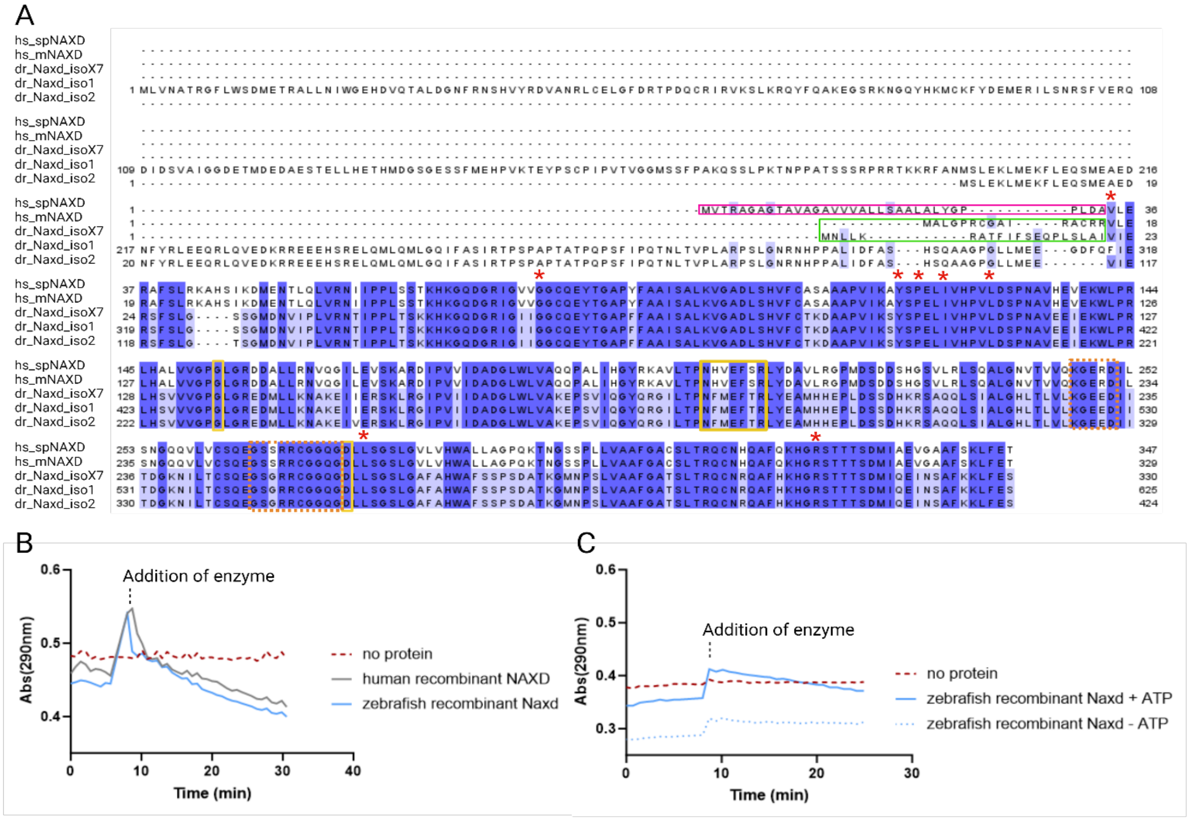

Supplementary Figure S1. (**A**) Multiple sequence alignment of human and zebrafish NAXD protein isoforms. Residues highlighted in dark, medium, and light blue are strictly conserved in all 5 protein sequences, in 4 of the 5 protein sequences, or in 3 of the 5 protein sequences, respectively. hs_spNAXD, human ER-targeted NAXD isoform (NP_ 001229810.1); hs_mNAXD, human mitochondrial NAXD isoform (NP_001229811.1); dr_Naxd_iso7, zebrafish Naxd isoform X7 (XP_005167521.1); dr_Naxd_iso1, zebrafish Naxd isoform 1 with the long N-terminus (NP_001315079.1); dr_Naxd_iso2, zebrafish Naxd isoform 2 (NP_001103590.1). Residues for which point mutations are known to cause NAXD deficiency in humans are indicated by red asterisks [1]. ATP and S-NAD(P)HX binding sites are indicated by dashed orange and yellow boxes, respectively (based on UniProt feature annotation). The predicted signal peptide of the human ER isoform is highlighted by a magenta box and green boxes indicate the predicted MTS sequences of the human and putative zebrafish mitochondrial isoforms. (**B**) Spectrophotometric monitoring of S-NADHX (500 µM) consumption in the presence of 1mM ATP and zebrafish recombinant Naxd (isoform X7, 10 µg/ml) or human recombinant mNAXD (NM_001242882.1 10 µg/ml) or in the absence of enzyme (negative control). (**C**) ATP-dependence of the zebrafish Naxd enzyme activity was verified by repeating measurements in the absence or presence of ATP.

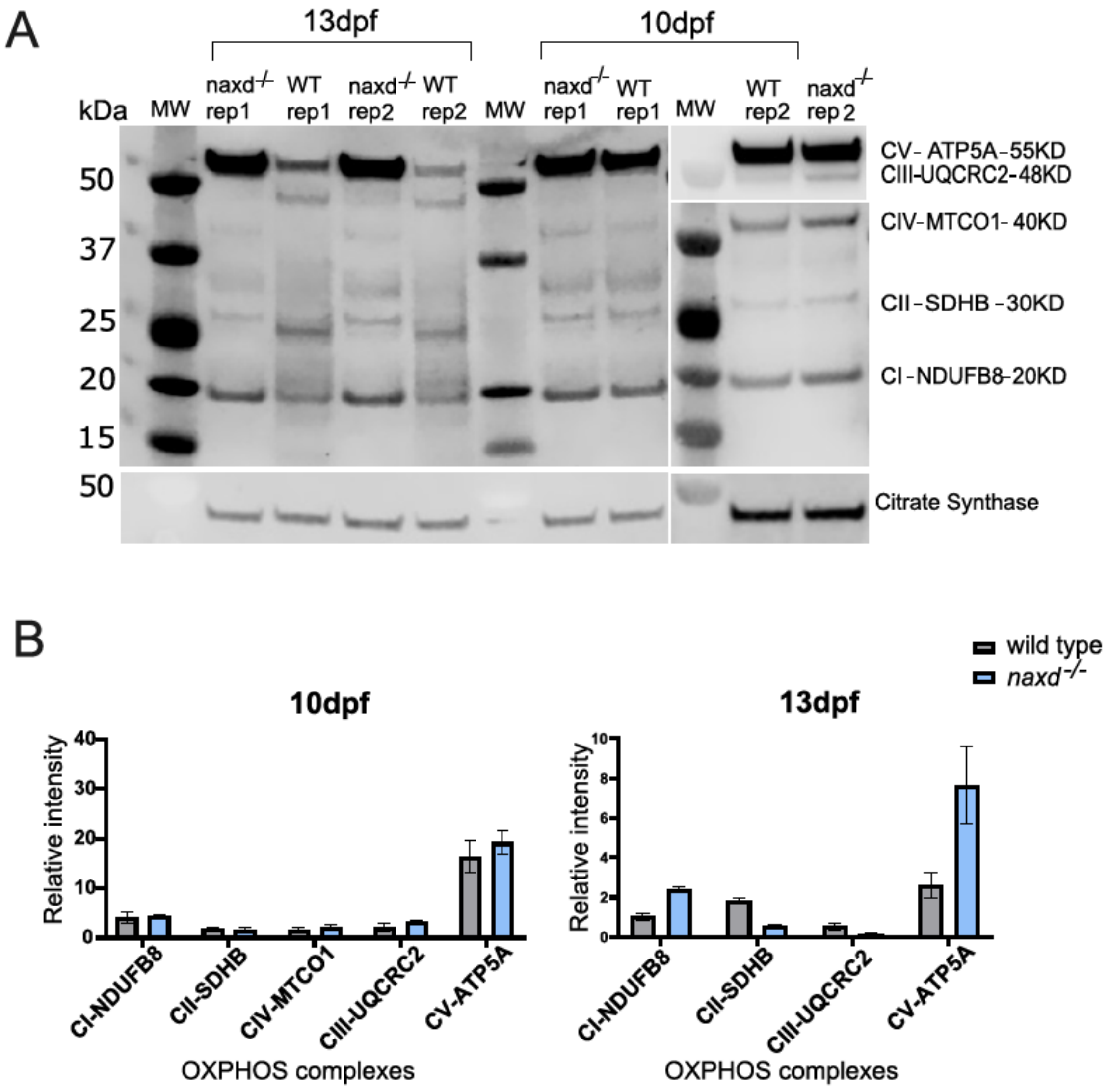

Supplementary Figure S2. (**A**) Western blot analysis showing the expression of mitochondrial oxidative phosphorylation (OXPHOS) complex subunits in whole-larvae lysates from wild-type (WT) and *naxd*$^{-/-}$ zebrafish larvae at 10 and 13 dpf. An antibody cocktail targeting representative subunits from complexes I–V was used. Two independent biological replicates (rep1 and rep2) are shown. The blot was cropped to adjust for differential exposure times required for the detection of individual subunits and the loading control. (**B**) Quantification of band intensities for all five OXPHOS complex subunits. Relative intensity indicates intensity values of the bands of interest normalized to the loading control (Citrate Synthase, expected MW 48.8 kDa), as measured using ImageJ software.

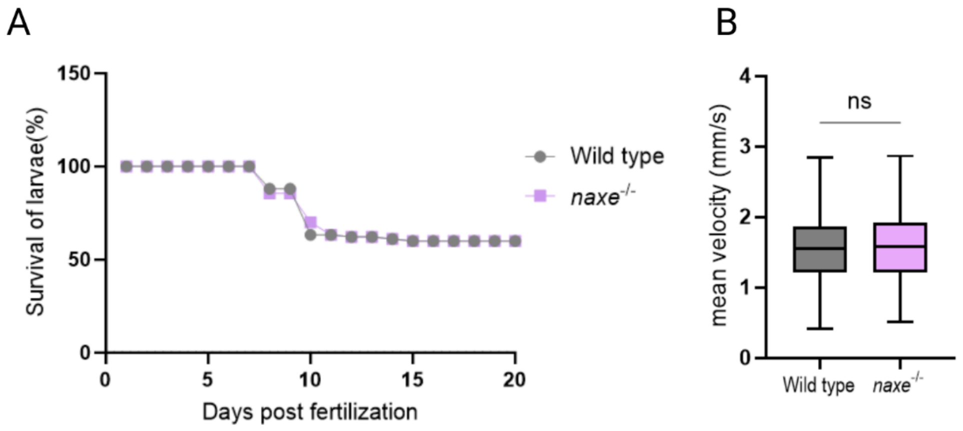

Supplementary Figure S3. Survival and locomotor activity analyses in *naxe*$^{-/-}$ larvae. (**A**) Survival analysis of *naxe*$^{-/-}$ larvae and wild-type siblings over 20 days. The results shown are representative of five independent survival experiments conducted each with approximately 20 larvae per genotype. (**B**) Locomotion behavior of 5 dpf *naxe*$^{-/-}$ larvae and wild-type siblings based on continuous tracking for 30 minutes in light condition (50% light intensity). Box plots represent means ± SDs of the mean velocity over 30 minutes (n=138 per genotype). ns, not significant (based on t-test).

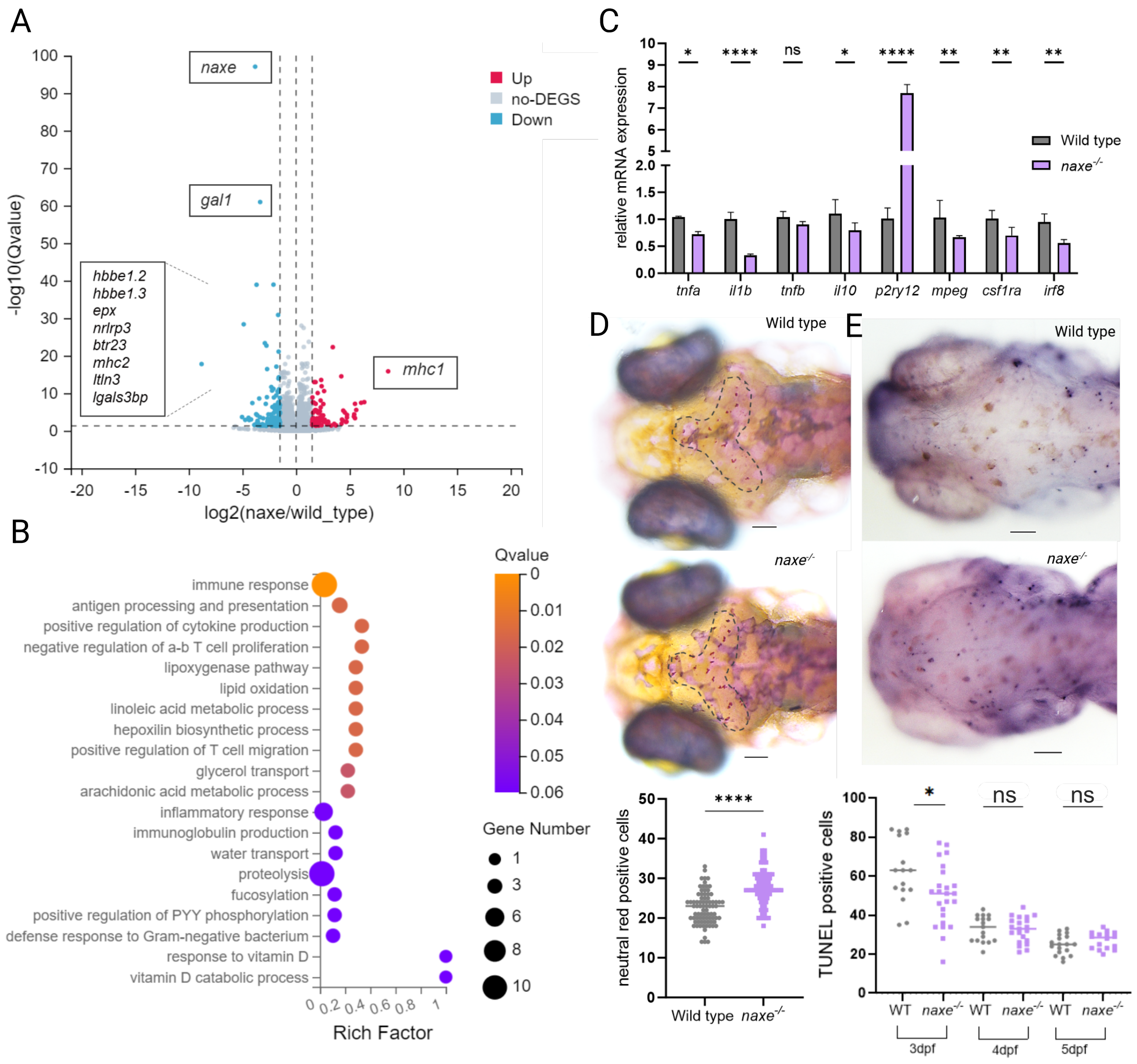

Supplementary Figure S4. Transcriptomics and imaging analyses in heads dissected from *naxe*$^{-/-}$ and WT larvae. (**A**) Volcano plot highlighting DEGs (mutant versus WT) with FC ≥ 1.5 and Qvalue ≤ 0.05. Significantly upregulated and downregulated genes are shown as red and blue dots, respectively; grey dots represent genes whose expression changes are below significance thresholds. (**B**) Rich factor plot of the GOp enrichment analysis performed on the DEGs. (**C**) mRNA expression levels of cytokines and microglia/macrophage markers in the heads of *naxe*$^{-/-}$ larvae (pink) relative to wild-type siblings (gray), based on qPCR analysis on RNA extracted from the heads of 5 dpf larvae. *actb1* was used as a reference gene. Data are means ± SDs from five biological replicates, each replicate consisting of a batch of 45 heads. (**D**) Neutral red stain (5µg/ml for 2 hours) of WT and *naxe*$^{-/-}$ larvae at 5 dpf; microglia/phagocytic cells are visible as red dots. The area used for phagocytic cell number determination is indicated by a dotted line. (**E**) TUNEL staining of WT and *naxe*$^{-/-}$ larvae at 3, 4, and 5 dpf; apoptotic cells are stained in dark purple. The scale bars represent 100 µm. Microglia and apoptotic cells were counted manually using ImageJ and results are shown in the bottom charts of panels **D** and **E**, with each dot representing one larva (n= 81 per genotype for neutral red staining, n= 16-24 per genotype for TUNEL assay) and horizontal lines representing the mean. Statistical significance was determined using unpaired t-tests (*p≤0.05, ** p≤0.01, ****p≤0.0001; ns, not significant).

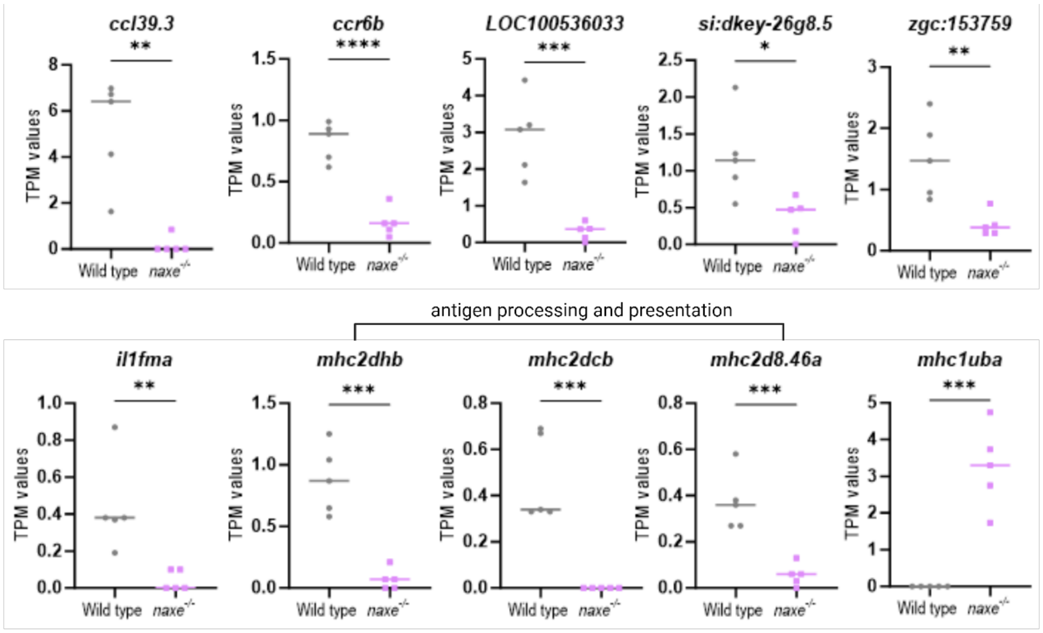

Supplementary Figure S5. Transcripts per million (TPM) values for each gene attributed to immune system processes in the GOp enrichment analysis of DEGs in *naxe*$^{-/-}$ versus WT samples, obtained from bulk RNAseq analyses. Data are shown as individual values (dots) for each of the 5 biological replicates and horizontal bars represent the mean. Statistical significance was determined using unpaired t-test (*p≤0.05, **p≤0.01, ***p≤0.001, ****p≤0.0001; ns, not significant).

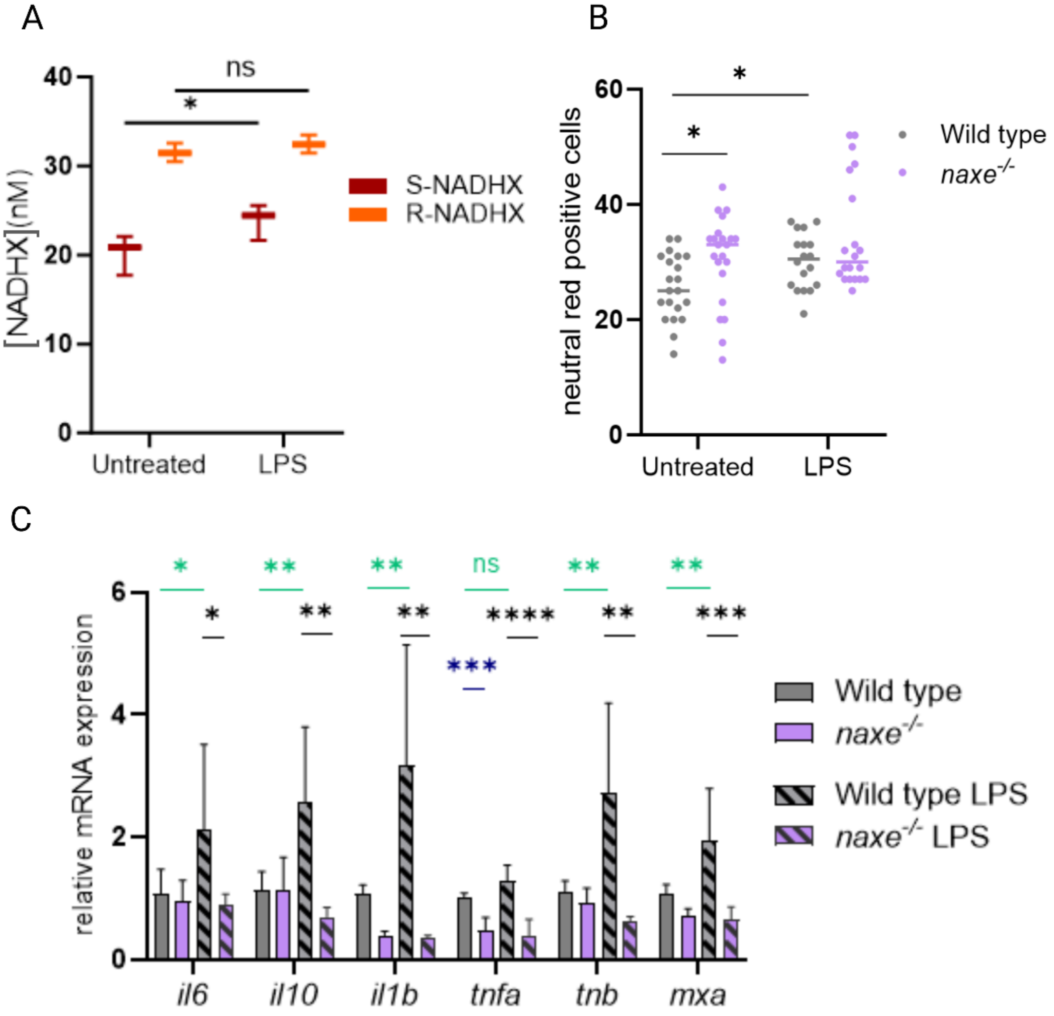

Supplementary Figure S6. LPS-induced immune response in 5 dpf wild-type and *naxe*$^{-/-}$ larvae. (**A**) S- and R-NADHX concentrations in extracts of 5 dpf *naxe*$^{-/-}$ larvae are shown as means ± SDs from three biological replicates, each replicate consisting of a batch of 35 whole larvae (raw data are provided in supplementary Table S3, experiment ZZURR). Statistical significance was determined with unpaired t-test (*$p \leq 0.05$). (**B**) Neutral red positive cell counts, determined with ImageJ in heads of 5 dpf larvae, are shown as individual values, each dot representing one larva (n=18-22 per genotype and condition). Statistical significance was determined with unpaired t-test (*$p \leq 0.05$). (**C**) Gene expression levels of cytokines in whole WT (gray) and naxe$^{-/-}$ (pink) larvae in basal conditions and upon LPS treatment (black stripes), determined by qPCR, are shown relative to the expression levels in untreated WT larvae. actb1 was used as a reference gene. Data are shown as means ± SDs from five biological replicates, each replicate consisting of a batch of 30 whole larvae at 5 dpf. Statistical significance was determined by two-way ANOVA with multiple comparisons test (*$p \leq 0.05$, **$p \leq 0.01$, ***$p \leq 0.001$, ****$p \leq 0.0001$). The following color code (asterisks) was used for the statistical comparisons: green, untreated versus LPS-treated WT larvae; black, LPS-treated WT versus LPS-treated *naxe*$^{-/-}$ larvae; dark blue, untreated WT versus untreated *naxe*$^{-/-}$ larvae. In the *naxe*$^{-/-}$ larvae, LPS treatment did not lead to statistically significant differences in expression compared to the untreated condition for any of the genes tested.

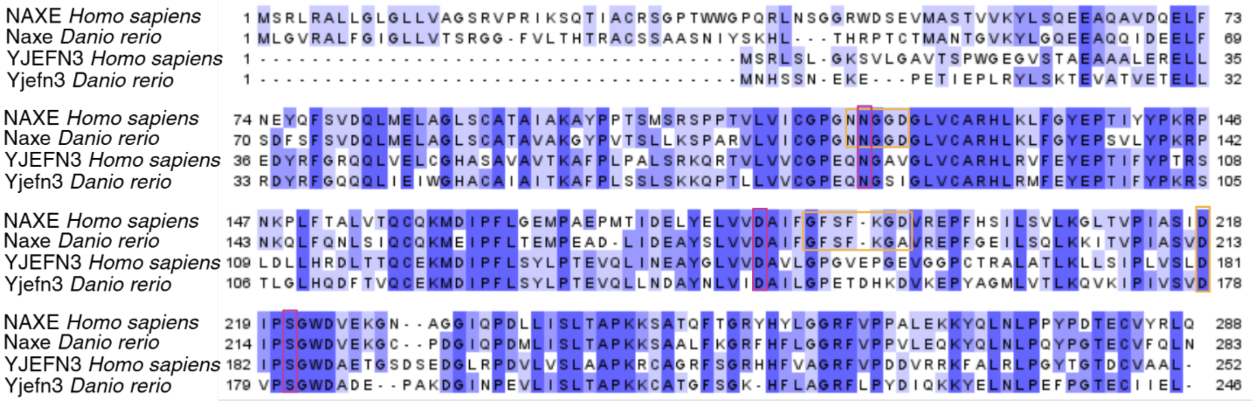

Supplementary Figure S7. Multiple sequence alignment of human and zebrafish NAXE and YJEFN3 proteins. Residues highlighted in dark, medium, and light blue are strictly conserved in all 4 protein sequences, in 3 of the 4 protein sequences, or in 2 of the 4 protein sequences, respectively. Human NAXE, NP_658985.2; zebrafish Naxe, NP_001002618.1; human YJEFN3, XP_054176892.1; and zebrafish Yjefn3, NP_001038308.1. Residues involved in NAD(P)HX binding and potassium binding in the NAXE protein (based on UniProt feature annotation) are highlighted by yellow and pink boxes, respectively.

Supplementary Figure Reference

1. Van Bergen, N.J., et al., *Clinical and biochemical distinctions for a metabolite repair disorder caused by NAXD or NAXE deficiency.* J Inherit Metab Dis, 2022. **45**(6): p. 1028–1038.
