# Supplementary material for: Unveiling Immune System Perturbations in Early Development Through Zebrafish Models of NADHX Repair Deficiency
Source: J Inherit Metab Dis. 2026 Feb 1;49(2):e70149. doi: 10.1002/jimd.70149 (PMC12861718; doi:10.1002/jimd.70149)
Supplement: Supplementary file 7 — Data S1: Supporting Information. [file JIMD-49-0-s005.pdf]

Unveiling Immune System Perturbations in Early Development through Zebrafish Models of NADHX Repair Deficiency

Myrto Patraskaki, Najmesadat Seyedkatouli, Lisa Schlicker, Marc O. Warmoes, Maria Lorena Cordero-Maldonado, Ursula Heins-Marroquin, and Carole L. Linster

Supplementary Data

Supplementary Materials and Methods

Locomotor behavior assays

Twenty-four hours prior recording, larvae were placed in 24-well plates containing 1 ml of Danieau's medium per well for habituation. The next day, each plate was placed in the DanioVision chamber and allowed to habituate for 15 minutes before starting the recording. The swimming behavior under normal light conditions (50% of total light intensity) of 5, 7, 10, and 13 dpf larvae was recorded for 30 minutes with the DanioVision system implemented with a temperature control unit set at 28 °C (Noldus Information Technology). EthoVision XT 11 and 17 software (Noldus Information Technology) was used to track the movements of individual larvae, expressed as mean swimming velocity (mm/s). For larvae older than 7 dpf, starvation was implemented for 24 hours before the recording. Each experiment was performed at least in three replicates with at least 10-12 larvae per genotype per replicate.

Neutral red staining and terminal deoxynucleotidyl transferase-mediated dUTP nick end labeling (TUNEL) assay

Embryos were maintained in 0.3X Danieau's solution without methylene blue and at 24 hpf, 0.003% PTU (1-phenyl 2-thiourea) (Sigma Aldrich catalog no P7629) was added to halt pigmentation and improve optical transparency. For the neutral red staining procedure, larvae were incubated with 5 µg/ml neutral red (Sigma Aldrich catalog no N7005) for 2 hours or 24 hours (for 5 dpf and 7 dpf larvae, respectively). After incubation, larvae were washed twice with 0.3X Danieau's solution without methylene blue containing 0.003% PTU, anesthetized with 0.008% tricaine methane sulfonate (MS222) (Sigma Aldrich catalog no E10521-10G) prepared in a phosphate buffer (pH 7.3-7.5), and

embedded in 3% methylcellulose for imaging purposes. Photos of individual larvae were taken using brightfield stereomicroscopy (Nikon SMZ25) and neutral red positive cells were manually counted using the counting tool of ImageJ (Version 1.53t).

For the TUNEL assay, performed as described in [1], 16-24 larvae at 3, 4, and 5 dpf were collected and fixed in 1 ml of 4% paraformaldehyde at 4°C. Single larvae were imaged in brightfield with an Olympus SZX7 stereomicroscope (1.5X). TUNEL positive cells were manually counted using the counting tool of ImageJ (Version 1.53t).

Metabolite extraction

Zebrafish larvae were maintained in 0.3X Danieau's solution and/or system water depending on the developmental stage. Before collection, larvae were washed once with distilled water and euthanized by hypothermia. Euthanized larvae were transferred to Precellys tubes pre-filled with 7 ceramic beads (1.4 mm, Qiagen, catalog no 1103955) (35 larvae at 5 dpf and 10 larvae at 10 dpf per tube). Samples were flash-frozen in liquid nitrogen, lyophilized overnight (FreeZone 2.5 Plus, Labconco, part number 7670020), and stored at -80 °C until metabolite extraction.

On the day of metabolite extraction, 600 µl of pre-cooled (4°C) extraction fluid (4:1 methanol:20 mM Tris, pH 8) containing thionicotinamide adenine dinucleotide (thio-NAD, 5 µg/ml) as internal standard (Sigma Merck, catalog no T7375) was added and the sample was homogenized using a Precellys24 homogenizer (Bertin Technologies) with 30 s cycles at 6000 rpm (0 to 5 °C, 3 cycles in total). The homogenate was transferred to a 1.5 ml Eppendorf tube. To recover a maximum amount of material, another 200 µl of extraction fluid were added to each Precellys tube and then mixed with the original homogenate, followed by a centrifugation at 21,000 g for 10 min at 4°C. After centrifugation, 625 µl of supernatant was transferred into a new 2 ml tube prefilled with 500 µl chloroform (4 °C) and 325 µl 20 mM Tris buffer, pH 8. The chloroform:methanol:Tris buffer mixtures were vortexed for 10 sec and centrifuged at 21,000 g for 5 min at 4°C to achieve phase separation. Next, 700 µl of the upper (polar) phase were filtered through PHENEX-RC 4 mm syringe filters and 600 µl of the filtered extracts were lyophilized overnight and stored at -80°C until analysis.

Targeted NAD(P)(H)(X) measurements by LC-MS

Lyophilized polar extracts were reconstituted in 50 µl of 50 mM ammonium acetate in MilliQ water, vortexed for approximately 20 seconds, spun down briefly, and transferred to LC-MS glass vials with micro-inserts. The analytical column (Polaris 180 Å C18-A, 3.0 x 150 mm, 3 µm) was maintained at 25°C. The autosampler was kept at 4°C and the injection volume was 20 µl. The mobile phases consisted of 50 mM ammonium acetate in MilliQ water (Eluent A) and acetonitrile (Eluent B) and a flow rate of 0.2 ml/min was used. Compounds were eluted with acetonitrile (ACN) using the following gradients: 0 min, 1% ACN; 5 min, 1% ACN; 22 min, 5% ACN; 27 min, 99% ACN; 30 min, 99% ACN; 30.5 min, 1% ACN; 40min, 1% ACN (re-equilibration step). Resuspended extracts were analyzed on two different LC-MS platforms, (1) an Agilent 1290 LC coupled to an Agilent 6560 Q-TOF MS equipped with a Dual Agilent Jet Stream ESI source or (2) a Vanquish Flex Binary coupled to an Exploris240 (Thermo Fisher). MS analysis on Agilent 6560 Q-TOF MS was performed using electrospray ionization in positive mode (+ESI, capillary voltage of 3.5 kV, nozzle voltage of 0 kV). The protonated molecules were monitored in high-resolution mode (slicer position: 5) and Extended Dynamic Range (2GHz) with the following Q-TOF MS conditions: drying gas temperature, 325 °C; drying gas flow, 11 l/min (nitrogen); nebulizer, 35 psi; sheath gas temperature, 350 °C; sheath gas flow, 11 l/min; fragmentor, 400 V; Oct RF Vpp, 750 V. Full scan spectra were acquired from m/z 100 to 1700 (1 spectrum/sec). External mass calibration was performed before the measurement of each set of samples. In addition, a reference solution was used for online mass correction during the acquisition. All data were acquired with Agilent Mass Hunter LC-MS/MS Data Acquisition (10.00). Manual peak integration was performed using Agilent Mass Hunter Profinder (the version used for each dataset is indicated in supplementary Table S2). MS analysis on Exploris240 was performed using electrospray ionization in positive mode (+ESI) with a spray voltage of 3.5 kV and the following HESI source settings: Sheath gas, 35; Aux gas, 7; Sweep gas, 0; Ion transfer tube temperature, 320 °C; Vaporizer temperature, 275 °C; S-lens RF level, 70.0. External mass calibration was performed before the measurement of each set of samples. Full scan spectra were acquired using lock mass correction (EASY-IC – RunStart) from m/z 100 to 1000 with a resolution of 60,000, setting the AGC target to *Standard*, the maximum IT to *Dynamic*,

and microscans to 2 with Xcalibur4.7 (Thermo Fisher). Peak integration and curation were done using TraceFinder 5.2 (Thermo Fisher) using an in-house compound database. All compounds were identified using standard compounds. S-, R-, and cyclic NADHX standards were synthesized and purified as described in [2]. For absolute quantification, standard compound samples were subjected to the same extraction process as the biological samples. Signal intensities of target metabolites were normalized to the internal standard thio-NAD and calibration curves of response factors were constructed in Excel. Absolute concentrations of target metabolites were calculated following linear regression analysis of the external calibration curves. Quantified values that had no raw signals were set to zero. Raw peak areas, normalized peak areas, and quantified values are provided in supplementary Table S3.

Western blot analysis of mitochondrial OXPHOS complexes

Total protein was extracted from whole zebrafish larvae using a lysis buffer (150 µl per 20 for 10-13 dpf larvae) containing 50 mM Tris-HCl (pH 7.5), 150 mM NaCl, 1% Triton X-100, 0.1% SDS, 1 mM EDTA, and 1x Roche (Ref. No. 05892791001) protease inhibitor cocktail. Larvae were homogenized using a Precellys24 homogenizer (Bertin Technologies) for three 30-second cycles at 6000 rpm (0–5 °C), followed by incubation on ice for 30 minutes. The lysates were then centrifuged at 16,000 *g* for 30 minutes at 4 °C to obtain the soluble protein fraction. Protein concentration in the supernatant was determined using the Pierce BCA Protein Assay Kit (Thermo Scientific, Cat. No. 23225) following the manufacturer's instructions. Equal amounts of protein (40 µg) were separated by SDS-PAGE using NOVEX 4–20% Tris-Glycine Plus WedgeWell precast protein gels (Invitrogen, Cat. No. XP04200BOX) and transferred to a PVDF membrane (Invitrogen, Cat. No. IB24002) using the iBlot2 dry blotting system. Membranes were blocked in 3% BSA (Sigma-Aldrich, Cat. No. A4503) in TBST for 1 hour at room temperature with agitation. Membranes were then incubated overnight at 4 °C with primary antibody (Total OXPHOS Rodent WB Antibody Cocktail, Abcam, Cat. No. ab110413, dilution 1:700, and and anti-Citrate synthase antibody, Abcam, Cat. No. ab96600, dilution 1:500). Following additional TBST washes (3 × 10 ml, 10 minutes each), membranes were incubated with the fluorescent secondary antibody (IRDye 800CW Goat

Anti-Rabbit, LI-COR, Cat. No. 925-32211, dilution 1:2500) for 1 hour at room temperature with agitation. After final washes (3 × 10 mL TBST), signals were detected using a LI-COR Odyssey FC imaging system. Band intensities were quantified using ImageJ software. The intensity of individual protein bands of interest were normalized to the corresponding loading control. Relative protein expression levels were then compared between the WT and *naxd*$^{-/-}$ samples.

Supplementary Results

Detailed description of gene expression analyses, neutral red staining, and TUNEL assays in *naxe*$^{-/-}$ zebrafish larvae

In a bulk RNA sequencing experiment on heads of 5 dpf larvae, where we detected 32.324 genes in total, 236 were differentially expressed (log2FC ≥ 1.5, Qvalue ≤0.05) between *naxe*$^{-/-}$ and WT siblings (Table S5). Among the DEGs, 152 genes were downregulated and 84 were upregulated in *naxe*$^{-/-}$ larvae compared to WT (Fig. S4A). Interestingly, 10 of the 13 most significant DEGs (Qvalue ≤ 1e-15) were related to the immune system (including galectin, inflammasome, and mhc genes). These 10 genes include galectins 1 and 3 (*gal1, lgals3b*), considered master regulators of immune responses and associated with neuroinflammation processes [3, 4], the NRLP12 inflammasome (*nrlp12*), and the major histocompatibility complexes I and II (*mhc1, mhc2*), well known for their roles in the immune response, intelectin 3 (*itln3*), with a key role in the pathogen response in zebrafish [5], and eosinophil peroxidase (*epx*), which partakes in host response processes [6] (Fig. S4A). Gene Ontology biological process (GOp) enrichment analysis, performed by considering all of the DEGs, also revealed the 'immune response' as the most significantly altered process (Fig. S4B). Furthermore, the majority of genes retrieved by the enrichment analysis participate in immune system processes and, interestingly, were consistently downregulated in *naxe*$^{-/-}$ larvae, except for one (*mhc1uba*) (Fig. S5). We also showed, by qPCR, reduced expression levels of a number of cytokine (*tnfa, il1b, tnfb, il10*) and macrophage signature (*irf8, mpeg1,* and *csf1ra*) [7-9] genes in the *naxe*$^{-/-}$ head

samples, that were not detected in the RNA sequencing experiments (Fig. S4C). Interestingly, the purinergic receptor *p2ry12*, associated more specifically with microglial cells [10] and whose signaling function is required for the engulfment of dying cells [11], was significantly upregulated in *naxe*$^{-/-}$ compared to WT siblings (Fig. S4C). Congruently, neutral red staining [12] revealed an increased microglial cell number or phagocytic activity in the head region of 5 dpf *naxe*$^{-/-}$ larvae compared to WT siblings (Fig. S4D). Although an increase in microglia number has been shown to positively correlate with developmental apoptosis [13], we did not find any significant differences in TUNEL-positive cell numbers between the heads of WT and *naxe*$^{-/-}$ larvae, at 3, 4 or 5 dpf (Fig. S4E). This suggests that neuronal cell death, at least the type detectable by TUNEL assay, is not increased during this developmental period in *naxe*$^{-/-}$ larvae.

Overall, these results suggested immune function dysregulation also in the *naxe*$^{-/-}$ larvae, and we further tested this hypothesis by analyzing the response of 4 dpf *naxe*$^{-/-}$ larvae to immune system activation by incubating them with LPS for 24 hours. S- and R-NADHX levels were only slightly increased in *naxe*$^{-/-}$ larvae after LPS treatment (Fig. S6A), while the damaged cofactors remained undetectable in the WT siblings also under this condition (not shown in Fig. S6A but data included in supplementary Table S3). Interestingly, although the number of microglial cells increased, as expected upon LPS treatment [14], in WT larvae, this was not the case in *naxe*$^{-/-}$ larvae (Fig. S6B). Finally, qPCR analyses revealed a complete lack of induction of expression by LPS of all tested immune response genes in *naxe*$^{-/-}$ larvae (in WT siblings, induction of gene expression was observed as expected; Fig. S6C). These results further support that *naxe* deficiency leads to impaired immune system development.

Supplementary References

1. Heins-Marroquin, U., et al., *Phenotypic assays in yeast and zebrafish reveal drugs that rescue ATP13A2 deficiency.* Brain Commun, 2019. **1**(1): p. fcz019.
2. Marbaix, A.Y., et al., *Extremely conserved ATP- or ADP-dependent enzymatic system for nicotinamide nucleotide repair.* J Biol Chem, 2011. **286**(48): p. 41246–41252.
3. Yaseen, H., et al., *Galectin-1 Facilitates Macrophage Reprogramming and Resolution of Inflammation Through IFN-beta.* Front Pharmacol, 2020. **11**: p. 901.

4. Srejovic, I., et al., *Galectin-3: Roles in Neurodevelopment, Neuroinflammation, and Behavior.* Biomolecules, 2020. **10**(5).
5. Ojanen, M.J.T., et al., *Intelectin 3 is dispensable for resistance against a mycobacterial infection in zebrafish (Danio rerio).* Sci Rep, 2019. **9**(1): p. 995.
6. Wang, J. and A. Slungaard, *Role of eosinophil peroxidase in host defense and disease pathology.* Arch Biochem Biophys, 2006. **445**(2): p. 256–60.
7. Shiau, C.E., et al., *Differential requirement for irf8 in formation of embryonic and adult macrophages in zebrafish.* PLoS One, 2015. **10**(1): p. e0117513.
8. Benard, E.L., et al., *Macrophage-expressed perforins mpeg1 and mpeg1.2 have an anti-bacterial function in zebrafish.* J Innate Immun, 2015. **7**(2): p. 136–52.
9. Ellett, F., et al., *mpeg1 promoter transgenes direct macrophage-lineage expression in zebrafish.* Blood, 2011. **117**(4): p. e49–56.
10. McKinsey, G.L., et al., *A new genetic strategy for targeting microglia in development and disease.* Elife, 2020. **9**.
11. Blume, Z.I., et al., *Microglia in the developing retina couple phagocytosis with the progression of apoptosis via P2RY12 signaling.* Dev Dyn, 2020. **249**(6): p. 723–740.
12. Herbomel, P., B. Thisse, and C. Thisse, *Zebrafish early macrophages colonize cephalic mesenchyme and developing brain, retina, and epidermis through a M-CSF receptor-dependent invasive process.* Dev Biol, 2001. **238**(2): p. 274–88.
13. Casano, A.M., M. Albert, and F. Peri, *Developmental Apoptosis Mediates Entry and Positioning of Microglia in the Zebrafish Brain.* Cell Rep, 2016. **16**(4): p. 897–906.
14. Tsarouchas, T.M., et al., *Dynamic control of proinflammatory cytokines Il-1β and Tnf-α by macrophages in zebrafish spinal cord regeneration.* Nature communications, 2018. **9**(1): p. 4670.
